# Molecular Dynamics Gives New Insights into the Glucose Tolerance and Inhibition Mechanisms on β-Glucosidases

**DOI:** 10.3390/molecules24183215

**Published:** 2019-09-04

**Authors:** Leon Sulfierry Corrêa Costa, Diego César Batista Mariano, Rafael Eduardo Oliveira Rocha, Johannes Kraml, Carlos Henrique da Silveira, Klaus Roman Liedl, Raquel Cardoso de Melo-Minardi, Leonardo Henrique Franca de Lima

**Affiliations:** 1 Laboratory of Molecular and Bioinformatics Modeling, Department of Exact and Biological Sciences (DECEB), Universidade Federal de São João Del-Rei, Campus Sete Lagoas, Sete Lagoas 35701-970, Brazil; 2 Laboratory of Bioinformatics and Systems (LBS), Department of Computer Science, Universidade Federal de Minas Gerais, Belo Horizonte 31270-901, Brazil; 3 Laboratory of Molecular Modeling and Drug Design, Department of Biochemistry and Immunology, Universidade Federal de Minas Gerais, Belo Horizonte 31270-901, Brazil; 4 Institute of General, Inorganic and Theoretical Chemistry (IGITC), Center for Molecular Biosciences Innsbruck (CMBI), Leopold-Franzens-Universität-Innsbruck, Innrain 82, 6020 Innsbruck, Austria; 5 Institute of Technological Sciences, Universidade Federal de Itajubá, Campus Itabira, Itabira 35903-087, Brazil

**Keywords:** β-Glucosidases, GH1, GH3, glucose tolerance, slingshot mechanism, allosteric channel, molecular dynamics simulation, free energy landscape, Poisson–Boltzmann surface area, grid inhomogeneous solvation theory

## Abstract

β-Glucosidases are enzymes with high importance for many industrial processes, catalyzing the last and limiting step of the conversion of lignocellulosic material into fermentable sugars for biofuel production. However, β-glucosidases are inhibited by high concentrations of the product (glucose), which limits the biofuel production on an industrial scale. For this reason, the structural mechanisms of tolerance to product inhibition have been the target of several studies. In this study, we performed in silico experiments, such as molecular dynamics (MD) simulations, free energy landscape (FEL) estimate, Poisson–Boltzmann surface area (PBSA), and grid inhomogeneous solvation theory (GIST) seeking a better understanding of the glucose tolerance and inhibition mechanisms of a representative GH1 β-glucosidase and a GH3 one. Our results suggest that the hydrophobic residues Y180, W350, and F349, as well the polar one D238 act in a mechanism for glucose releasing, herein called “slingshot mechanism”, dependent also on an allosteric channel (AC). In addition, water activity modulation and the protein loop motions suggest that GH1 β-Glucosidases present an active site more adapted to glucose withdrawal than GH3, in consonance with the GH1s lower product inhibition. The results presented here provide directions on the understanding of the molecular mechanisms governing inhibition and tolerance to the product in β-glucosidases and can be useful for the rational design of optimized enzymes for industrial interests.

## 1. Introduction

β-Glucosidases are enzymes with great importance for many industrial processes, such as the production of wine [1], animal feed [2], and biofuel [3,4]. In the second-generation biofuel production, they act in synergy with endoglucanases and exoglucanases for the conversion of lignocellulose into fermentable sugars [5,6]. β-Glucosidases catalyze the last and limiting step, converting cellobiose into two molecules of glucose [7]. However, most β-glucosidases are inhibited in high concentrations of glucose, which provides an accumulation of cellobiose [8,9]. Moreover, accumulated cellobiose may cause inhibition of other enzymes that catalyze the first reactions of cellulose hydrolysis [10,11]. Hence, β-glucosidases have been considered key enzymes in the second-generation biofuel process, and they have been the target of many studies that aimed to elucidate the inhibition mechanisms and also propose modifications that could improve the catalytic activity, thermostability, and tolerance to glucose inhibition [3,12,13,14,15,16,17,18,19,20,21,22].

Most β-glucosidases have been classified into the glycoside hydrolase families 1 (GH1) and 3 (GH3) [20]. β-glucosidases from GH1 family have been described as more efficient for biofuel production due to a higher resistance to glucose inhibition and a more conserved structure, which allowed more studies of beneficial mutations [6,13,20,23]. Giuseppe et al. [12] argued that GH1 β-glucosidases are tenfold to 1000-fold more glucose-tolerant than GH3 due to their active site be located in a deep and narrow cavity, while the GH3 β-glucosidases present a shallow pocket. For instance, they compare the three-dimensional structures of *Humicola insolens* GH1 β-glucosidase (here called HiBG), hence more resistant to glucose inhibition, and the *Aspergillus aculeatus* GH3 β-glucosidase (here called AaBG), thus, more susceptible to glucose inhibition. They supposed that the difference in the inhibitory behavior of these two enzymes could be a consequence of the difference in the accessibility of glucose to the catalytic site. HiBG presents an active site at 17 Å of depth, while AaBG at 11.3 Å. However, an explanation of why the accessibility mechanism would be more relevant for the product than to the substrate entrance is still missing.

Yang et al. [13] suggested that relative binding affinity of glucose to some sites at the entrance and middle of the substrate channel modulates the glucose dependence and regulates the effects of product inhibition in the GH1 enzymes. Using site-directed mutations, they proposed that three residues 228, 301, and 302 of a marine metagenome GH1 β-glucosidase (Bgl1A) could be crucial to glucose tolerance. They detected that mutations in analogous residues (H228T and N301Q/V302F) of a homologous non-tolerant β-glucosidase (Bgl1B) led to glucose tolerance. By inspection virtual docking analysis, they have advocated that this effect would be due to the favoring of the glucose binding to these sites considerably distant from the catalytic cleft. The same study has pointed the position 228 as presenting higher significance on the tolerance acquiring effect. The secondary effect of the other two would be due to their proximity of the first. The authors also have suggested that this region is an allosteric subsite (AS).

In a more recent study, the comparison between crystallographic structures of tolerant and non-tolerant GH1 β-glucosidases has shown that the tolerant seems to have the region close to the AS prolonged on a kind of allosteric channel (AC), providing an alternative thermodynamically favorable binding site that is not the same as catalytic cleft [24]. Moreover, a computational approach detected mutations at the residue 228 and the AS/AC neighborhood from the metagenomic Bgl1B lead to structural signatures more similar to glucose tolerant β-glucosidases [25]. In another study, Mariano et al. [3] analyzed 21 glucose-tolerant β-glucosidases and detected a consensus sequence of 22 amino acids in the substrate channel. Using molecular docking of cellobiose at a modeled structure of a *Thermoanaerobacter brockii* β-glucosidase [26], they detected that the residues W122, N166, E167, C170, L174, Y299, E355, W402, E409, and W410 perform contacts with the ligand. These residues are located in the substrate channel, with a considerable part of them on the AC/AS neighborhood, which highlights the importance of this region for glucose tolerance.

In the mentioned studies, a dynamic depiction of the possible mechanisms in which the AS/AC region would guide the glucose to exit or thermodynamically compete with its binding at the catalytic cleft was never described. In this way, a considerable set of studies seems to point to glucose tolerance/inhibition paradox. The confront between how favorably glucose binds at the catalytic cleft against how favorably it binds at alternative regions appears to be the answer. However, these studies are based on the analysis of static structures or the functional interpretation of site-directed mutagenesis. Hence, to better understand the expulsion mechanisms, the dynamics aspects of protein and ligand, as well the dynamics behavior of the water energetic along with the active site, must be considered [27]. In this sense, molecular dynamic simulations (MD) are a very contumacious way to get dynamic and energetic information of biological systems at the atomic scale at the same time and consider the solvent effects explicitly.

In this study, we performed a set of MD simulations for the GH1 β-glucosidase of *Humicola insolens* (HiBG) and the GH3 β-glucosidase of *Aspergillus aculeatus* (AaBG). We analyzed the cellobiose (substrate) and glucose (product) mobility in each system, protein–ligand interactions among different regions of the active site, and we calculated the ligand-free energy landscape (FEL) for the protein active site. We also analyzed the role of the water favorability at different active subsites by the confront of grid inhomogeneous theory (GRID) and Poisson–Boltzmann surface analysis (PBSA) for representative protein conformations. The results presented here provide the first dynamic depiction of the glucose withdrawal mechanism in GH1 and an explanation about the lower adequacy of this mechanism in GH3 enzymes. They supply guidelines to the understanding of the molecular mechanisms governing inhibition and tolerance to the product in β-glucosidases of industrial interest. We hope they provide means for obtaining new biotechnologically optimized enzymes, with an imminent impact on the improvement in second-generation bioethanol production.

## 2. Results

### 2.1. Equilibration and Conformational Sampling from the MD Sets

**Figure 1:**
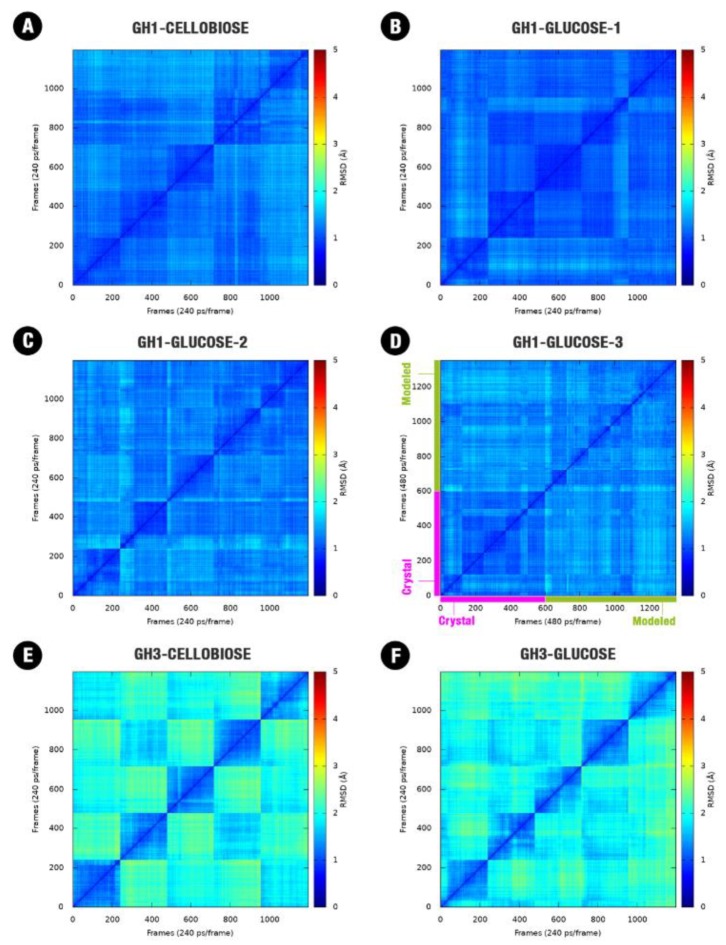
Two-dimensional root-mean-square deviation (RMSD) plot for the combined five trajectories (60 ns each one) for each one of the starting systems: (**A**) GH1 (HiBG)–Cellobiose complex, (**B**) GH1–glucose complex with glucose starting from the crystallographic pose, (**C**) GH1–glucose complex with glucose starting from the modeled pose, (**D**) GH1–glucose crystallographic complex (magenta) and the complex starting from the modeled pose (green). (**E**) GH3 (AaBG)–Cellobiose complex, (**F**) GH3–glucose complex. RMSD post the alignment of all the frames and considering just the backbone atoms (Cα, C, O, N).

To probe the equilibration and sampling convergence for different MD simulations and systems, we constructed two-dimensional root-mean-square deviation plots (2D-RMSD; Figure 1). We observed convergence in the protein dynamics for GH1s in the presence of the substrate and the product (Figure 1A–D). Additionally, the protein conformational sampling at the GH1-glucose system does not change significantly independent of the glucose starting position (Figure 1B–D).

**Figure 2:**
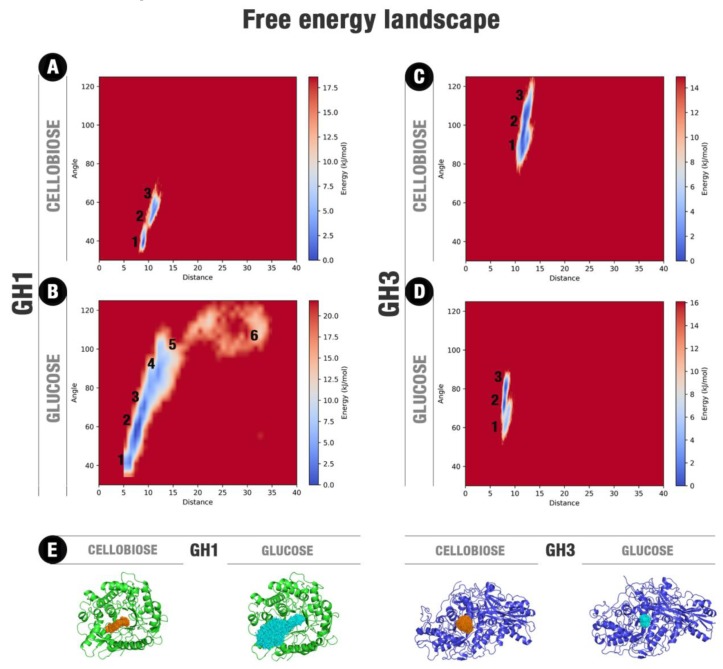
Free energy landscapes (FEL) for cellobiose and glucose at their respective and individual complexes with GH1 and GH3. (**A**) HiBG complexed with cellobiose, (**B**) HiBG complexed with glucose. This FEL was depicted considering together the respective GH1–glucose-1 and GH1-glucose-2 sets of simulations. The respective starting points at the simulations GH1–glucose-1 and GH1-glucose-2 are at the regions 3 and 1, (**C**) AaBG complexed with cellobiose, (**D**) AaBG complexed with glucose. The distance is relative to the geometric center of each ligand and the catalytic E378 (in GH1) or H121 (in GH3). The angle is formed by these two geometric centers and the geometric center of each protein. (**E**) Superposition of the ligand poses around all the respective trajectory ensembles for each system. Protein average frames are shown in green (HiBG) and blue (AaBG) cartoons. All frames of ligand positions for cellobiose (orange) and glucose (cyan) were overlapped.

For AaBG, protein conformational convergence is less evident in the presence of cellobiose or glucose (Figure 1E,F). In some cases, we can notice a conformational superposition between different simulations (with an RMSD < 2 Å) still at the relaxing phase (not shown). While the GH1 enzymes are globular and relatively compact, presenting the mobile loops with size considerably smaller than the GH3 enzymes, these are considerably higher in terms of mass, present an elongated shape and bigger loops (Figure 2E). All these aspects allow a higher movement amplitude for the most mobile parts of the AaBG when compared to HiBG. This higher amplitude for the GH3 movements led to conformational convergence at the same scale as GH1.

The ligand movements present a higher convergence and reduced mobility at the GH3 enzyme than at GH1, even though the mobility of GH3 is higher than GH1 (Figure 2, Figures S2–S5, Figure S10). At this lower mobility condition, the glucose on the AaBG active site and along our MD simulations has established hydrogen bonds mainly with the polar residues at the −1 subsite, overall residues D73, K170, H171, and D261, beyond hydrophobic interactions with M226 and W262 (Figure S4, Figure S9, and Tables S4–S6).

The comparative principal component analysis (PCA) between the movements of the protein and the ligand shows that the ligand freedom inside the GH1 enzyme is higher than in GH3. Moreover, we observed a more upper coupling between the ligand and protein movements in the GH1 when compared to the GH3 (Figure S10). This was observed for GH1 simulations with cellobiose and glucose. For the last ligand, such effect accentuates so its mobility than culminates with its escaping from the active site in two simulations with different starting conditions.

We also performed the free energy landscape (FEL) analysis considering the ligand space inside the actives site to verify the comparative energetic freedom of cellobiose and glucose on each system (Figure 2). It is noteworthy, the considerably high freedom of glucose inside the GH1 enzyme compared to the other systems. For HiBG complexed with glucose (Figure 2A) and AaBG complexed with cellobiose and glucose (Figure 2C,D), the ligands cannot access positions significantly far from the initial point. On the other hand, for HiBG complexed with glucose, the ligand can access several regions of the energy landscape, consonant with the higher glucose mobility in this system, as observed in Figure 2E and Figures S2–S5. The gain in translational mobility in this system culminates with the access of the energetic region six on Figure 2B, depicting the glucose withdrawal.

### 2.2. MD Shows Glucose Releasing for HiBG (GH1)

**Figure 3:**
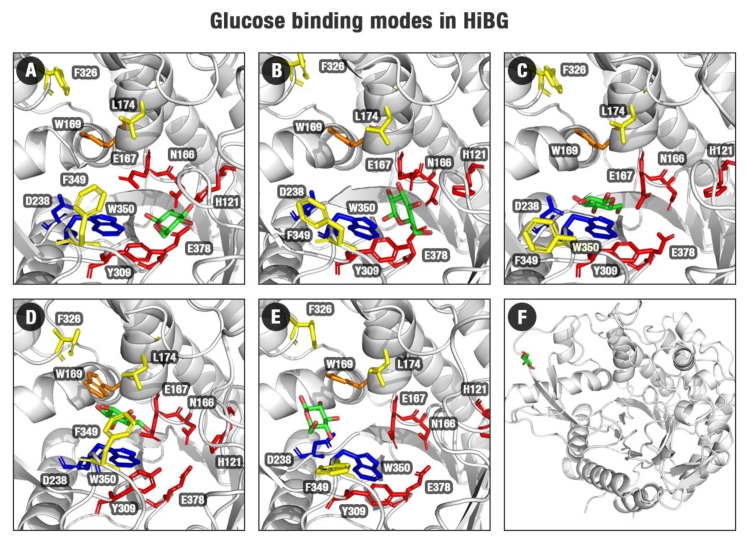
Binding modes for HiBG in complex with glucose of the GH1–glucose FEL (Figure 2B) for the regions 1 (**A**), 2 (**B**), 3 (**C**), 4 (**D**), 5 (**E**), and 6 (**F**). Protein structures are shown in gray, subsite −1 (red sticks), subsite +1/+2 (yellow sticks), D238/W350 (blue sticks), W169 (orange sticks). The poses (selected from the minima at the FEL from Figure 2B) show a probable exit path for glucose from the HiBG active site.

For the MD of the GH1–glucose complex, we observed the complete ligand releasing to the bulk in two respective individual trajectories. The first one starting from the modeled catalytic pose (with total duration of 90 ns up the complete glucose exit) and the other starting from the crystallographic one (with glucose initially at the intermediary site, packed against residues W169, L174, Y180, F349, and W350; the last with the default duration of 60 ns). For the remaining replicates, glucose presented itself significant freedom to occupy different subsites (Figure S2), but it was positioned with considerably higher frequency at the middle portion of the substrate channel between subsites −1 and +1 (region 2 at the FEL from Figure 2B, and pose in Figure 3B). This is an intermediary region, and its relatively higher occupancy shows the high facility of glucose to pass through that from one subsite to the other.

By the profiles of Figure 2A,B and the superposition of cellobiose and glucose poses in Figure 2E and Figures S2 and S3, we observed that ligand translation (and consequent glucose exit) along the GH1 active site is biased in terms of angle relative to the catalytic cleft and the protein geometric center. There is a preferential path from where the ligand moves and, eventually, escapes (Figure 3).

In pose one (the closest to the active site), glucose interacts with the polar −1 subsite (N166, E167, H121, and E378, Figure 3A, Figure S6, Tables S1–S3). Then, it leaves the catalytic cleft and goes to the middle of the substrate channel at the minimum two (the most statistically populated position according to Figure 2A,B FEL, Figure 3B). It is worth noting that, while on the less energetically favorable catalytic pose, the water-mediated hydrogen bonds prevalence, at the more favorable pose two, the participation of these water-mediated bonds decreases and the sites for direct hydrogen bonds increase (Figure S7, Tables S1 and S2). Concerning the behavior of glucose at the GH3 catalytic cleft, the participation of the direct hydrogen bonds is higher than the water-mediated hydrogen bonds (Figure S9, Tables S4 and S5). This indicates that direct interactions with the active site wall and water exclusion are important to determine the favorability of the glucose retaining at different sites at the β-glucosidases here studied.

Subsequently, glucose is trapped by hydrophobic interactions with residues between the middle and the entrance of the channel, where this ligand interacts mainly with W350, and in a minor scale with L174, W169, W436, F349, and Y181 (Figure 3C,D, Figure S7, Tables S1–S3). This state is corresponding to region three in Figure 2B FEL. This set of hydrophobic residues is denoted in the literature as gatekeeper residues, acting as bottlenecks for the substrate channel, limiting the entrance and exit of molecules [2]. Their roles have been correlated with glucose tolerance [2,3]. Finally, glucose is released from the hydrophobic region, mainly due to interactions with D238 (Figure 3E,F). This residue represents the analog to position 228, described as the allosteric site (AS) in [13,25,26]. Our studies suggest this amino acid has significant importance for glucose releasing in glucose-tolerant β-glucosidases, in a process herein described as “slingshot mechanism”, which will be better presented in the next sections.

### 2.3. APBS vs. GIST vs. Ligand Fitting: Differences on the Electrostatic Distribution, Water Activity and Stereochemical Adjustments Around the Active Sites of Hibg and Aabg and Ligand Dynamics 

**Figure 4:**
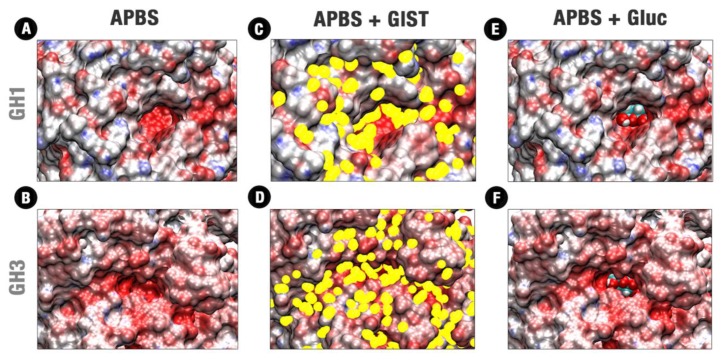
Confront between APBS, and GIST data and the glucose fitting in GH1 and GH3 representative poses at the catalytic cleft. (**A**,**B**) APBS respectively for HiBG at the pose representative of the minimum 1 in Figure 2B and AaBG at the pose representative of the minimum 2 in Figure 2C; (**C**,**D**) APBS and GIST results respectively for the previous pose of HiBG and AaBG (**E**,**F**) APBS and glucose fitting respectively for the previous pose of HiBG and AaBG. APBS scale in red, white, and blue corresponding to ψ values of −20.00:0.00: +20.00, respectively. GIST results are shown in yellow dots for water interacting centers with g^v^(O) ≥ 10.00 units of the bulk density and ∆G^v^_solv_ ≥ +3.0 kcal/mol. Glucose is shown in cyan spheres with carbon atoms in cyan, oxygens in red, and hydrogens in white.

The APBS and GIST analyses of the decoys recovered from the MD FELs (Figure 2) show a high negative electrostatic potential as well a significant trend to favorable water retention at the catalytic cleft of both enzymes (Figure 4A,B,D). This is consonant with the presence of the two catalytic acids, the polar residues of the −1 subsite and the necessary adequation of this position to the polar substrate. However, a distinction in topology and distribution of polarity is found at the catalytic cleft and neighborhood between the two enzymes. The catalytic cleft at HiBG is located at the bottom of a 17–18 Å deep tunnel, with decreasing polarity up to the nonpolar loops at the surface. On the other hand, AaBG presents a constrict catalytic cleft surrounded by a flat and shallower intermediary region, around 6 Å distance from the catalytic residues, equally charged and hydrophilic.

The GIST analysis of the same decoys shows that both catalytic pockets are also centered with high potential for water exclusion (Figure 4C,D). Considering just sites with occupancy g^v^(O) ≥ 10 units of the bulk density, only unfavored water sites (∆G^v^_solv_ ≥ +3 kcal/mol) are recovered in our analyses for both enzymes. This indicates that the significant losses in terms of entropy (for the strongly retained water molecules) as well as in water–water interactions are, in general, not enthalpically compensated enough by the new water–solute interactions at this same site. This is also in agreement with the known importance of the solvent exclusion on the binding mechanisms in the active sites [28].

Despite the similarities mentioned above, the differences in topology and polarity distribution also induce distinctions on the GIST recovered profiles on the active sites of both enzymes. The differentially distributed polarity of the HiBG active site and the width of its catalytic cleft tend to distribute the unfavorable water retention sites majorly on the sides, forming hydrophobic paths that go from the bottom to the surface (Figure 4C,D). On the other hand, the shallower catalytic cleft of the AaBG tends to be uniformly filled with unfavorable hydration sites for all its extension, the same considering the flat and equally confined region around it and the apolar loops at the surface.

**Figure 5:**
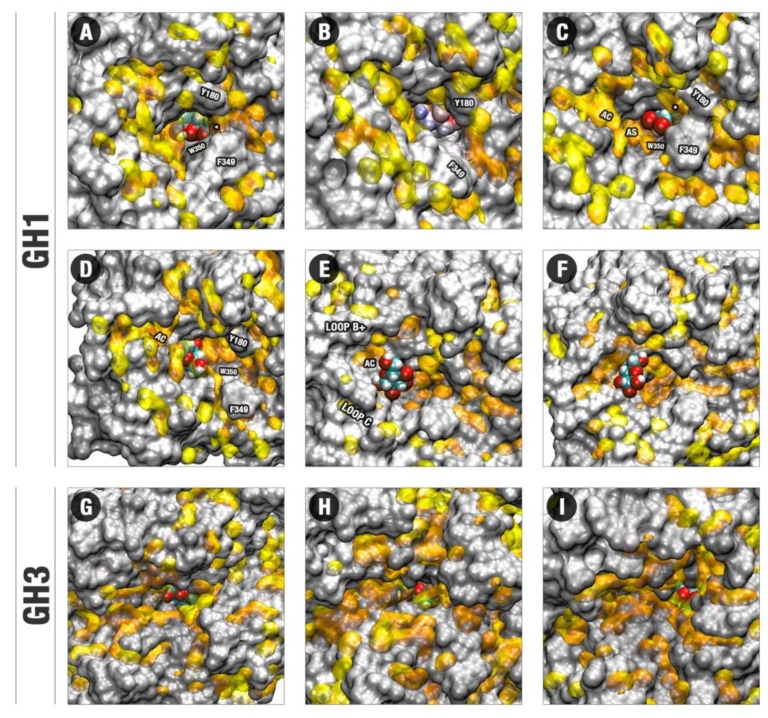
Conformations recovered by the FEL profiles of the glucose positioning in GH1 and GH3, colored by the GIST data. (**A**–**F**) GIST results and glucose positions occupancy at the respective FEL regions 1–4 and two different samples of region 5 in GH1 in Figure 2B. (**G**–**I**) The analog profiles for the FEL respective regions 1–3 in GH3 in Figure 2C. GIST positions are shown in yellow transparent surfaces for water-interacting centers with g^v^(O) ≥ 10.00 units of the bulk density and ∆G^v^_solv_ ≥ +3.0 kcal/mol. Glucose, shown in spheres in all the figures, is colored with carbons in cyan, oxygens in red, and hydrogens in white (except for B, which was colored by APBS). Residues involved in the establishment of the hydrophobic cage and regions important for the glucose escaping in GH1 are highlighted. AS: Allosteric site. AC: Allosteric channel. A white asterisk (*) is used to point the site for water exclusion confined by the hydrophobic cage between Y180, F349, and W350. The same figure colored by APBS and GIST is available in the Supplementary Materials (Figure S13).

Figure 4E,F compares the respective allocations of glucose at the HiBG and AaBG post catalytic poses. Glucose is relatively loose inside the deeper catalytic cleft of the GH1 enzyme, contacting the unfavored water regions of Figure 4C majorly by the ligand extremities (Figure 5A). On the other hand, glucose is more stereochemically fitted by the polar fence at the shallower catalytic cleft of AaBG. This high stereochemical adequacy agrees with a higher enclosure of glucose by the polar residues of the −1 site in the GH3 (Figure S4) that in the GH1 enzyme (Figure 3A,B and Figure S2). The allocation of glucose at the GH3 catalytic cleft is also more superposed to the unfavored hydration sites at this cleft in Figure 4D in all their extension (Figure 5G–I).

Figure 5 suggests that the distinctions of topology, distribution of charge and unfavorable hydration sites, as well of stereochemical adequation are straightly related to the dynamic differences of glucose on the GH1 and GH3 enzymes. The movement of glucose in a direction outside the active site in the HiBG occurs according to this ligand, loosely bound at the catalytic cleft, diffuses along the hydrophobic paths and between the different hydrophobic subsites connecting the bottom to the surface (Figure 5A–F). In Figure 5A,B, the respective decoys corresponding to the minima 1 and 2 in Figure 2B and the poses in Figure 3A,B are depicted. It can be noted that from pose A to B, glucose diffuses from the catalytic cleft through a hydrophobic path that brings this ligand in proximity to the apolar residues W350, F349, and Y180 (hydrophobic bottleneck). Then, subtle reorientations of the side chains (majorly F349) promote a kind of hydrophobic cage, in which glucose tends to be retained, being also impaired to return by the same path to the catalytic cleft (Figure 5C). The decoy here analyzed corresponds to the minimum of region three from Figure 2B and the pose in Figure 3D. At this position, glucose is orientated in a way that facilitates the establishment of hydrogen bonds with the D238 residue (AS, Figure S7, Tables S1 and S2). At the next decoy (corresponding to the local minimum four in Figure 2B and the pose in Figure 3E), glucose is completely displaced to this AS (Figure 5D). It can be noticed at this pose that glucose is positioned on a site with significant potential for water exclusion, indicating that the binding to the AS is favorable due to the hydrogen bonds with D238 and hydrophobic effects. Continuous to this allosteric site, there is an allosteric channel (AC) delimited by side chains from the loop C and an insertion of the loop B not usually present at the normal non-tolerant GH1s, here called loop B+. This allosteric channel is filled by unfavorable hydration sites, forming a hydrophobic path that connects the AS to the external environment. In Figure 5E, depicting a next decoy from the region 5 of Figure 2B FEL, it can be noted that subtle movements of the residues at the loops C and B+ promote at the same time a higher opening of the AC and a significant reduction at the potential for unfavorable water retention at the region of the AS. In this way, the hydrophobic effect at this region diminishes, glucose becomes free to unbind the AS and follow by the enlarged AC. However, the AC never loses its hydrophobicity completely. In Figure 5F, depicting a subsequent decoy on the region five from Figure 2B, it can be noted that a set of hydrophobic patches remains, so that glucose maintains itself at the proximity, establishing occasional CH/π hydrophobic interactions. This effect, in turn, draws back the return of this ligand inside the active site. On the subsequent events at the escaping MDs, glucose gradually unbinds this channel, or it is guided through the same channel to outside, in both cases resulting in the exit depicted in Figure 3F.

For GH3, the unfavorable water sites also form narrow hydrophobic channels, some of them being, in principle, compatible with possible guidance of glucose outside the active site in a similar way as on GH1 (Figure 5G–I). However, in all representative decoys, this ligand presents higher stereochemical adequacy to the polar catalytic cleft compared to the GH1 system. Also, the region where the ligand binds in this cleft has higher superposition with unfavorable hydration sites. The catalytic cleft and its immediate surroundings can be considered as hot spots of unfavorable water sites on the GH3 pocket. In this way, the permanency of glucose at the catalytic cleft on the non-tolerant enzyme seems to be favored both by the direct protein–ligand interactions and by the hydrophobic water exclusion effects (in consonance with the reduced number of water-mediated hydrogen bonds depicted for this system at Table S5), so that glucose remains tightly bound at the GH3-glucose system along all the MDs set.

The same analysis concerning the protein–cellobiose MD simulations shows similar behavior to the respective GH1–glucose and GH3–glucose complexes concerning the water retaining sites, but with crucial distinctions relative to the presence of the two glucose monomers establishing interactions with different subsites at the same time (Figure S8). For GH1, considering together the two respective glucosides (the reducing and the non-reducing), a comparable trend can be seen where the ligand occupies the same major subsites of the hydrophobic paths as in the glucose simulations. However, a critical difference is precisely the fact that the interactions of the non-reducing and the reducing glucosides with the active site occur at the same time, reinforcing each other. This impairs the complete withdrawal of the substrate from the active site, despite the relative mobility of the same. This same mobility, in the case of cellobiose inside the GH1 active site seems to allow a better synergic exploring of the active pocket environment by the two glucosides. For instance, in Figure S11C, it can be noted that the movement of cellobiose allows a synergic binding of the ligand reducing glucoside at the AS and of the non-reducing close to the hydrophobic cage. Both regions are hot spots for hydrophobic interactions according to the GIST results, as already described. The synergic interaction of both glucosides at the two hot spot regions impairs the ligand to follow the AC or unbind the active site on a similar way that glucose when interacting with the AS.

For the GH3, the additional reducing glucoside is considerably less fitted to the active site wall and less superposed to the water exclusion sites than the non-reducing or the individual glucose molecule (Figure S11D–F). This agrees with the high rotational mobility of the reducing glucoside inside GH3 compared to the non-reducing one, as depicted in Figure 2E (third image) and Figure S5. It is also in consonance with the lower number of contacts depicted for the same reducing glucoside in Figure S8 and Tables S4–S6. Beside this, the presence of this extremity and its contacts with the external loops (even though sparse) reduce the average enclosure of the catalytic fence and of the −1 subsite residues around the non-reducing extremity, resulting in a higher mobility also for this extremity (as can be noted in Figure S5) compared to the individual glucose in Figure S4.

In this way, the analysis of the data related to APBS, GIST and stereochemical fitting here presented suggests an active pocket more suited to the residence of the substrate than the product on the tolerant GH1 here studied, while there is apparent qualitatively opposite behavior for the non-tolerant GH3.

### 2.4. Evidence of the Participation of the D238 Residue and Neighborhood on the Glucose Catapulting Outside the GH1 Enzyme

**Figure 6:**
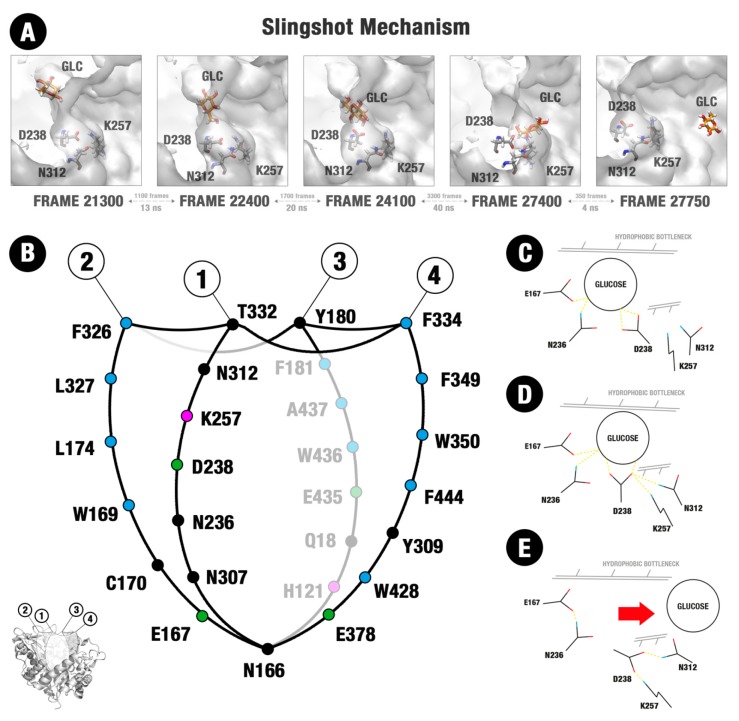
Structure of the substrate channel of HiGB and the mechanism of glucose release. (**A**) Five snapshots of the MD simulation that illustrate the slingshot mechanism and residues involved. (**B**) Structure of the substrate channel organized into four sectors (1–4). A set of hydrophobic residues is located in the entrance of this channel, mainly in the sectors 2, 3, and 4. Circles represent apolar (blue), polar neutral (black), polar positively charged (magenta), and polar negatively charged (green) amino acids. (**C**–**E**) Amino acid residues involved in the slingshot mechanism: E167, N236, D238, K257, and N312.

An interesting aspect is noted concerning the dynamics of D238 and its neighbor residues at the two simulations in which glucose escapes completely from the active site. When glucose is far from D238, this residue tends to make polar interactions majorly with the dyad K257 and N312 (Figure 6A, first snapshot). As glucose is guided to the residue D238 by the hydrophobic paths and the protein movements, as well other auxiliary polar interactions, the residue D238 interrupts its interactions with the polar dyad and establishes hydrogen bonds with the glucose (Figure 6A, second and third snapshots). In the next snapshots, the diffusion of glucose in the direction of the AC and posterior withdrawal of the active site is accompanied by a movement of D238 back to the K257-N312 dyad (Figure 6A, fourth and fifth snapshots). The sequence of movements resembles a slingshot catapult, as if the D238 caught the glucose and then, being attracted again to its polar partners, it threw the ligand in the direction of the AC region. Although such movement has been observed at the two simulations in which glucose escaped from the active site, presenting a relatively small sampling, its apparent relationship with the withdrawal mechanism has not gone unnoticed. Beyond that, it involves a region known for its importance in glucose tolerance [13,25,26].

In this way, the possible implications of such movement on the intrinsic mechanism of glucose escaping from the tolerant GH1 enzyme will be included in the discussion section. 

## 3. Discussion

### 3.1. Tolerance and Inhibition are Dependent on a Set of Topological and Physical-Chemical Distinctions Between the Respective GH1 and GH3 Active Sites

Many aspects of glucose tolerance in GH1s are not fully understood up to now. Previously published studies attribute the tolerance of HiBG (and other glucose-tolerant GH1 β-glucosidases) to a higher difficulty of glucose to have access to the catalytic cleft compared to the non-tolerant enzymes, as AaBG (GH3) [12]. The issues described as limiting for the glucose access are the higher depth of the substrate channel and the presence of gatekeeper hydrophobic residues at the tunnel entrance. However, it is not clear why these aspects would not impair the access of the substrate to the catalytic region equally.

Another hypothesis points to the presence of an alternative allosteric binding site (AS) specific to glucose that would impair the access of this ligand to the catalytic cleft, facilitating its withdrawal from the active pocket. It is not evident why this alternative site would be more acceptable to glucose than to cellobiose binding and what would be the dynamic/energetic mechanisms that could facilitate the driving of glucose from the catalytic position to the same site and from the AS to outside the pocket. Without such final liberation of glucose from this site, the retention of this ligand at the same would favor more a transglycosylation process than tolerance [13].

Our computational results suggest a mechanism of glucose tolerance based on a combination of the aforementioned attributes. Data here presented shed light on how these characteristics combined allow a ligand launching mechanism intrinsic to the GH1 and more adapted to glucose than to the substrate exit. The mechanism involves a set of particular characteristics of the tolerant GH1 active site distinct from the non-tolerant GH3, each one of them in concurrence with the previous issues pointed in the literature: (i) A different substrate/product stereochemical fitting relationship, favoring more the substrate than the product fitting on the GH1 pocket and, apparently, the opposite in GH3. This differential fitting results on significantly higher mobility of glucose and greater responsivity of this ligand to the protein dynamics inside the GH1 active site. In this way, glucose is easily guided by the protein dynamics to the sites that favor its withdrawal, (ii) a different distribution of charge and hydrophobicity at the GH1 and GH3 active pocket which tends to generate water constraining paths in GH1, along which glucose tend to be hydrophobically driven to the exit zones, while in GH3 there is a favoring to the residence of this ligand on the catalytic cleft. Considering cellobiose, the water-constraining paths in GH1 seem to create zones that facilitate the hydrophobic adjustment of this larger ligand along different regions of the active site in simultaneous. In this way, the same mechanism seems to contribute more efficiently to the substrate permanency and the product escaping, and (iii) the action of an allosteric site (AS) continuous to an allosteric channel (AC) typical of tolerant GH1 enzymes. This allosteric site, together with the physical-chemical attributes, promotes a kind of slingshot mechanism. In this mechanism, glucose is first guided to the AS. The small polar residue at this site catches glucose and, boosted by the opening of the AC and by a set of local interactions, catapults this ligand outside the active site. 

### 3.2. The High Mobility of Glucose Inside the GH1 Active Site, Corroboration with the Sparse Electronic Density of HiBG

In our MD sets, glucose inside the HiBG’s active site has demonstrated significantly energetic accessibility to different regions (Figure 2B) corroborated by its considerable mobility (Figure 3, Figure S2, Figure S10B) and culminating with withdrawal in two independent MDs starting by two different poses (Figure 3F). This high mobility agrees with the observed significantly lower stereochemical adjustment of glucose to the catalytic cleft of the GH1 enzyme compared to GH3 (Figure 4E,F). This set of clues agrees with the experimental evidence of the sparse electronic density, as well the atypical position of glucose at the crystallographic structure of the HiBG-glucose complex compared to other non-tolerant GH1s (Figure S1). The typical GH1-glucose crystallographic complexes present a well solved electronic density (describing clearly all the heavy atoms with σ = 1.5) at the −1 site (Figure S1A,B). However, glucose in HiBG is located close to the exit (at the hydrophobic cage visualized at our MD studies) and with a scarcer electronic density, hardly describing just the regions around the carbons 5 and 6, the oxygen at the position 1 and the carbon-oxygen pair at which would be the position 2 (Figure S1C,D). This evidence, per se, indicates higher mobility of glucose inside the HiBG compared to usual non-tolerant GH1s, confirming our computational data. However, we must stress the possibility that the electronic density described as from glycerol at the usual glucose position in HiBG should be from a still less solved (and so, most mobile) glucose at this site (Figure S1D). Considering the sparse electronic density that has allowed the attribution of the glucose identity at the hydrophobic cage at this structure, it cannot be attributed to the uniqueness of the definition of the electronic density at the −1 site as if in fact from glycerol or a second (and most mobile) glucose molecule. This absence of uniqueness persists even if we carry out an analysis with less strict σ criteria (σ ≤ 1, not shown). The presence of less solved glucose at this site, together with a subtly better solved at the hydrophobic cage, would be in conformity with the relative FEL values at the two sites in Figure 2 and with the lower stereochemical adjustment at the −1 subsite depicted in Figure 4E.

### 3.3. The Importance of the Hydrophobic Subsites at the Exit and the Water Activity Modulation on The Glucose Withdrawal

An unclear point in the importance of the hydrophobic gatekeeper regions described in the study of Giuseppe et al. [12] is: if the hydrophobic residues restrict the entrance of glucose in the substrate channel, why would they not restrict the entrance of cellobiose? Or even, would they be responsible for retention of glucose in the middle of the substrate channel, which could cause the enzyme inhibition? 

Our computational data show that the zones propense to ligand binding due to water exclusion effects in GH1 do not restrict just to the hydrophobic residues at the entrance of the channel. These zones form true paths that connect from the bottom of the substrate channel (the catalytic cleft at the −1 subsite) to the exterior region. In the case of the less stereochemically fitted product (glucose), the movement of the protein and the modulation of the hydrophobicity along these paths is easily able to conduct this ligand along them up to the AS/AC region and finally to the exit. In the case of the two glucoside rings, the same movement of the protein leads this ligand to orientations that allow the simultaneous interactions of both of those regions highly propense to water exclusion at the same time that reinforces the interactions at each site in a cooperative way. The same physical-chemical characteristics of the water-constrained paths mediate the paradoxical effect of facilitating the glucose diffusion to outside and cellobiose permanency at the active site.

It is interesting to highlight that a significantly important region at the hydrophobic mediation of the glucose withdrawal and cellobiose permanency, the hydrophobic cage composed by the residues W350, F349, W169, L174, and Y180 (Figure 3C,D, Figure 5D, Figure S2, Figure S3, Figure S11B,C), has a substantial corroboration from the literature. This is the region where the electronic density of glucose (even than sparse) is solved in HiBG. The residues W169 and L174 are described in the literature as promoters of glucose tolerance [3]. The W350 is a conserved residue in GH1 enzymes. The establishment of the hydrophobic cage is strongly determined by movements and packing of the residues F349 and Y180. The F349 is located on a small insertion at the loop C, found on the HiBG but not at the usual non-tolerant GH1s. Considering the positions topologically analogous to these two hydrophobic residues and based on the five GH1 PDBs used as a comparison in Figure S1, we usually find hydrophobic residues, but relatively smaller, such as alanine or leucine at the analogous position to 180 and valine or methionine on the analogous to 349. While the characteristic remains, the volume and coverage at these hydrophobic regions can be a target in the improvement of glucose tolerance in usual GH1 enzymes.

For the GH3 enzyme, the catalytic cleft seems to represent a hotspot for water exclusion that, together with higher stereochemical adjustment to glucose, seems to be a determinant factor in the product permanency on the catalytic site in our simulations. Future studies of rational design of more glucose-tolerant GH3s can take the enhancing of the hydrophobicity at the flat region immediately outside the catalytic cleft and promote a higher water exclusion effect compared to the cleft itself. This could be of interest due to the higher usual catalytic activity of this class of enzymes [29,30]. Another possibility could be the shortening of the external loops, once their approximation contributes to bringing the −1 subsite residues on the imprisonment position around glucose (Figure S4). This position seems to contribute to the high stereochemical fitting of the product at the catalytic cleft and the high constraining of unfavorable water molecules at the apo state (Figure 4D,F, Figure 5G–I), both contributing to the product retention.

Due to the idiosyncratic polarity characteristics of carbohydrates (i.e., being polar ligands, but with CH/π interactions accounting to their interactions with protein-binding sites), it is not surprising that the ability to interact with hydrophobic paths has an influence on their residence or escaping in β-glucosidases [31].

### 3.4. The Importance of an Allosteric Site/Channel and a Slingshot Mechanism for the Glucose Withdrawal

Our MD data confirm the role of D238, the analogous position of the residue 228 previously described in [13,25], as an allosteric sub-site responsible for impairing the occupancy of glucose at the catalytic cleft. More than this, the data here presented depicts the first dynamic/energetic description of the mechanism by which this site promotes such effect.

The fundamental role of this position at the glucose immobilization and driving to the exit seems to be dependent of previously described hydrophobic paths, of its continuity to an allosteric channel (AC) formed between the loops C and B+ (Figure 5D–F), as well as local interactions at its neighborhood that promote a kind of slingshot, catapulting glucose outside (Figure 6). The effect of mutations that enhance the access to this site on the increase of glucose tolerance, as described in [13,25], can be better understood in the face of the present data. Additionally, it is important to highlight the participation of loop C in the formation of the AC from where glucose finally escapes in our simulations. The loop C topology has been already described as a determinant for glucose tolerance in GH1 enzymes [3]. Moreover, in loop C are located the W350 and F349 residues, both fundamental to the establishment of the hydrophobic cage.

Beyond the role of the hydrophobic paths, other polar interactions seem to be relevant for the guidance of glucose up to the AS position at the D238 residue (Figure 6B–E). If we divide the catalytic pocket of the GH1 enzyme into a set of sections (based on the statistically relevant sites depicted in Figures S6–S9 and Tables S1–S6 and the active site topology), we obtain the Section 2, Section 3 and Section 4 majorly hydrophobic and the Section 1 (where the D238 residue is located), majorly polar (Figure 6B). Section 2, Section 3 and Section 4 participate more actively on the establishment of the water constraining zones that create a hydrophobic way to conduct glucose from the catalytic cleft to the hydrophobic cage. However, along the glucose path to the hydrophobic cage and from there to the AS, this ligand is also guided by hydrophilic interactions with residues from the Section 1 and neighbors, overall, the catalytic E167 and N236 (Figure 6C–E). Between them, N236 has been already described as presenting significant importance in the glucose tolerance mechanism [32].

**Figure 7:**
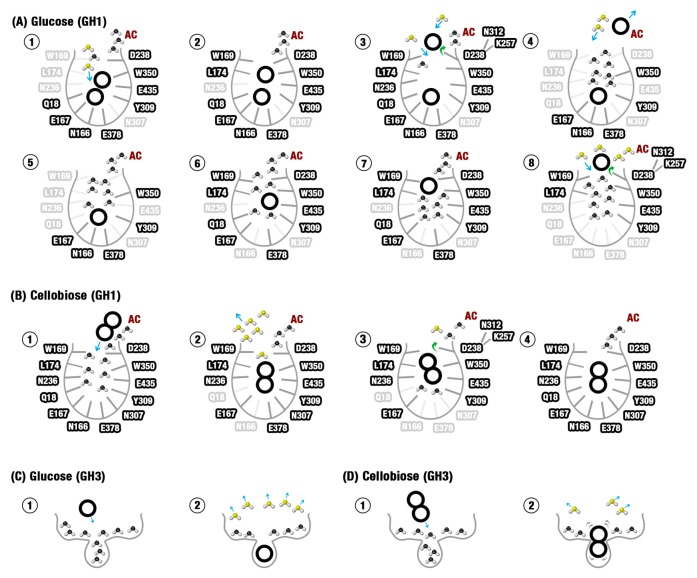
The sequence of molecular events that lead to glucose tolerance at the GH1 enzyme and inhibition at the GH3. (**A**) Slingshot mechanism influences the glucose tolerance of the HiBG GH1 enzyme. Figure 7A1–8 shows the most probable sequence of events (based on the computational issues here recovered), from the hydrolysis to the final elimination of the non-reducing glucose. (**B**) Cellobiose cannot so easily escape from the HiBG substrate channel. The sequence of events depicted in B1–4 is similar to the events described for glucose. However, the higher set of synergic interactions involving the protein and the two glucose rings in cellobiose draws back the substrate. (**C**) At the shallower and more charged pocket from the AaBG GH3 enzyme, the catalytic cleft is more stereochemically fitted to glucose. Besides this, the same constricted cleft tends to retain a higher set of unfavorable water molecules that are more efficiently liberated with the glucose binding. (**D**) The relatively small cleft of AaBG is less stereochemically suited to cellobiose than to glucose, resulting in higher ligand mobility and less unfavorable water elimination. AC: Allosteric channel. Unfavorable waters (black) are energetically constrained water molecules, favorable waters (yellow) depict water molecules liberated or energetically relaxed, bold countered residue names depict residues involved in interactions at the represented state, transparent residue names are residues not involved in interactions at the represented state.

The results here presented shed light on the molecular mechanisms that lead to the glucose tolerance/inhibition paradox between GH1 and GH3 enzymes. They allow the proposal of a detailed description of the mechanism of selective glucose escaping in glucose-tolerant β-Glucosidases (here called slingshot mechanism) and why the same mechanism is not facilitated in an intolerant GH3. We expect that new industrially promising modifications on GH1 and GH3 enzymes can be formulated in the future. Figure 7 presents a model of the tolerance/inhibition mechanisms here proposed, including all the physical-chemical elements.

## 4. Materials and Methods 

### 4.1. Protein and Ligand Structures

The crystallographic structures of β-glucosidase A from *Humicola insolens* in complex with glucose (PDB: 4MDP, with a 2.05 Å resolution) [12], as well β-glucosidase 1 from *Aspergillus aculeatus* in complex with glucose (PDB: 4IIG, 2.30 Å resolution) and cellobiose (PDB: 4IIH, 2.00 Å resolution) [33] were both collected from the Protein Data Bank [34].

For 4MDP structure, glucose is not found on the catalytic cleft, where it use to be found on other GH1-glucose crystallographic complexes (as in PDB: 3VIJ, PDB: 4PTX, PDB: 3WH6, PDB: 2JIE, PDB: 2O9T, PDB: 2E40, the last, complexed with gluconolactone), but packed between the residues W169, L174, F349, and W350, on an intermediary position between the catalytic cleft and the active site exit, henceforth cited as the intermediary site (IS, Figure S1A). On the other hand, close to the usual position of the catalytic cleft (depicted by the E167 and E377 catalytic acids), there is crystallographic glycerol in HiBG (Figure S1B). While the electronic density of the PDB:4MDP glucose at the IS is significantly sparse compared to the glucose on the catalytic cleft on the other structures (Figure S1C), suggesting that a glucose transition site, the electronic density of HiBG crystallographic glycerol is approximately as high as that from the glucose usually found at this position (Figure S1C,D). Considering the relative chemical similarities between glycerol (a poly-alcohol) and carbohydrates, as well as the catalytic cleft, the usual position of glucose in GH1 enzymes, we have used two different starting structures for our GH1 MD simulations: The crystallographic one (with the original glucose starting at the experimental IS position) and a prepared one, with glucose at the catalytic position. This last structure was built by structural alignment with the *Neotermes koshunensis* β-glucosidase A complexed with glucose (PDB: 3VIJ, resolution of 1.03 Å), conserving the glucose from this last complex and the protein from the PDB: 4MDP one. The alignment was carried out considering just the protein backbone (RMSD of 0.6 Å) using the PyMOL software. For the GH1-cellobiose complex, once there is no experimental structure of the HiBG complexed with cellobiose, we have carried out just the structural backbone alignment with the PDB: 3VIK (*N. koshunensis* β glucosidase in complex with cellobiose, with a resolution of 1.10 Å and alignment RMSD of 0.611 Å), conserving the cellobiose from the PDB: 3VIK structure. For the simulations of the GH3 systems, both the protein structures and the respective glucose and cellobiose at the catalytic clefts of each original PDB were properly used.

For all the respective systems, the protonation states of the histidines were estimated by the H++ online server using the default salinity and dielectric parameters (respectively, 0.15 M, the internal dielectric of 10 and 80) and pH 7.00 [35]. 

### 4.2. Molecular Dynamics Simulations

All the systems were solvated with a TIP3P cubic box with a 12 Å padding (measured from the outermost point of the protein) using the plugin tleap of the AmberTools package [36]. Na^+^ and Cl^−^ ions were added until neutrality and ionic strength of 0.15M. In each system, the protein, water, and ions were described by the ff99SB AMBER force field, while the respective carbohydrates were described by GLYCAM06 [37,38]. All the simulations were carried out at the NPT ensemble, keeping the temperature at 300 K by a Langevin thermostat and the pressure at 1 bar with an isotropic implementation of the Berendsen barostat. Periodic boundary conditions were carried out, using a cutoff of 10 Å for the nonbonded interactions, and solving the long-range electrostatic interactions by particle mesh Ewald (PME). The respective 1–4 interactions for protein and carbohydrate were scaled according to the specific default politics of each force field. All the simulations were executed using as a numeric integrator the AMBER16 software [36], a numeric time step of 2 fs and treating the hydrogen coordinates by the SETTLE constraint algorithm.

After the above-mentioned system prepare, a minimization/relaxing protocol was carried out consisting of: (i) 1000 steps of energy minimization by conjugate gradient, (ii) 300 ps of NPT pre-relaxation with harmonic restraint for protein and carbohydrate, (iii) 200 ps NPT relaxation removing the carbohydrate restrains, (iv) 200 ps of NPT relaxation without restrictions to the side chains of the residues 5 Å around the ligand, (v) 200 ps of NPT relaxation with no restrictions for the whole residues around the ligand, (vi) 10 ns of unprecedented NPT pre-production. This protocol was carried out in five replicates for the respective starting structures (after their respective protonation, solvation, and ionization) from each one of the five systems: HiBG-cellobiose, HiBG-glucose (crystallographic position), HiBG-glucose (catalytic position), AaBG-cellobiose, and AaBG-glucose. Henceforth, these specific starting systems will be called as GH1-cellobiose, GH1-glucose-1, GH1-glucose-2, GH3-cellobiose, and GH3-glucose, unless previously mentioned in the opposite. After that, from each one of these respective five replicates per system, an independent productive MD protocol was carried out with the reinitializing of the velocities for a 300 K compatible distribution, the carrying out of a 60 ns NPT simulation (300 K and 1 bar) and saving the coordinates at every 4 ps. Altogether, five completely independent sampling simulations were carried out (from the minimization/relaxation to the productive step) for each respective GH1–cellobiose, GH3–cellobiose, GH3–glucose system and ten completely independent for the GH1–glucose system (considering the two different starting points for glucose at this system). For the GH1–glucose-2 system, the specific simulation in which the glucose releasing has turned evident was extended up to 90 ns (30 ns more) in order to allow the complete exit of this ligand. In this way, and taking together all the independent simulations, a sampling corresponding to 300 ns of productive MD (encompassing 75,000 frames) was collected for each respective GH1–cellobiose, GH3–glucose and GH3–cellobiose system and a 630 ns one (corresponding to 157,700 frames) for the GH1-glucose.

We also carried out GIST (grid inhomogeneous solvation theory) designated MD simulations just for the protein chain of the representative conformers selected from the protein–ligand FEL profiles. Firstly, we extracted the ligand and solvated the protein on an octahedral box using the AmberTools package with a minimum distance between the box edge and the protein of 12 Å [39]. We used the same TIP3P water model and described the protein–water system with the same ff99SB force field and used the same H++ estimated protonation states previously applied at the sampling MD. In sequence, we equilibrated and simulated the systems at respective 100 ns of productive MDs, but with 50 kcal^.^mol^−1^ coordinate restraints for the entire protein, as required by GIST analysis [40] at AMBER18 [39]. Moreover, we did describe the ions explicitly for the GIST designated MDs, having neutralized the system by a uniform plasma algorithm, as implemented in AMBER18. This last approach is recommended, in order to reduce the solvent complexity and allows the use of the respective references for bulk density and water–water interaction energy as the one of pure TIP3P simulations (see Section 4.6 below). All the remaining NPT and general MD conditions were maintained according to the sampling MD methodology above described. The coordinates were saved at every 100 ps for the GIST analysis itself. 

### 4.3. MD Trajectories Analysis

2D-RMSD plots (all the frames against all the frames) related to the protein backbone were carried out using the cpptraj plugin from AmberTools [36] to check convergence along the different simulations for each different system. For the GH1-glucose system, in special, this analysis was carried out considering in separate simulations the two respective starting orientations of the glucose as these two systems together.

Principal component analysis (PCA), considering the protein backbone as the ligand heavy atoms were carried out using WORDOM [41]. In both cases, the covariance matrix calculation, diagonalization, and estimation of the eigenvector projections were made aligning the protein backbone of all the MD frames to the average structure. Principal components (PC) of proteins represent internal (conformational) movements, while the ligand PC is dominated by the translational and rotational motions of the ligand inside the active site. 

To analyze the pattern of the different contact types between the ligand and protein (direct hydrogen bonds, water-mediated hydrogen bonds, and hydrophobic contacts) along the different active site sub-regions, we used the LIGPLOT software [42]. For hydrogen-bond calculations, we considered 2.7 Å as the maximum hydrogen–acceptor distance and 3.35 Å as the maximum acceptor–donor one. Non-bonded contact parameters were performed using 2.90 Å as the minimum-contact distance and 3.90 Å as the maximum-contact one.

### 4.4. Ligand–Protein Free Energy Landscape Estimations

To get a glimpse of the product/substrate energetic freedom inside and outside (for the system in that the ligand is released) the active site of each protein, we estimated the Gibbs free energy landscape (FEL) from the pose density using the plugin GMX from Gromacs 2.1 [43]. This algorithm uses a histogram method to estimate the relative ΔG at different subregions of a 2D sampling space of two collective variables (respectively r_1_ and r_2_), descriptors of the phenomena of interest (in this case the ligand position along the trajectories) [44]. The ΔG value at each subregion of the 2D plot is estimated by the Boltzmann distribution probabilities along this space according to the (1):

(1) $$\Delta G\left(r_{1},r_{2}\right)\;=\;-k_{b}T\;lnP\left(r_{1},r_{2}\right)$$

For the purpose of this study, r_1_ was set as the cartesian distance between the respective geometric centers from the ligand and a reference residue at the deeper part of the catalytic site, where r_2_ was the angle involving these two respective geometric centers and the geometric center of the protein. For the GH1 protein studied here, the reference residue was the catalytic E378, while for GH3 it was the H121 at the −1 site.

Finally, from the FEL minima, the representative poses of the ligand pathway along the active site were selected using a homemade algorithm that located the more densely populated grids along different subregions of the 2D histogram and the respective trajectory frames that more approximated from these values (in r_1_ and r_2_).

### 4.5. Poisson–Boltzmann Surface Area

To get insights into the mechanisms of energetic complementarity of the protein active site to the substrate and product at the representative poses accessed along the MD simulations, both the electrostatic potential distribution along the protein surface (with special attention to the active site) and the water energetics along the same active site were estimated and compared.

In order to have a glimpse of the electrostatic potential distribution, we estimated the Poisson–Boltzmann surface area, with the integrated use of the online versions of the PDB2PQR and the adaptive Poisson–Boltzmann solver (APBS) tools, respectively [45,46,47,48]. Basically, the PDB2PQR software was used to generate the input files for the PBSA calculations with APBS, i.e., the pqr file, containing the PDB atomic coordinates as well the atomic charges and radii at the respective beta and gamma columns, and an input file containing the grid dimensions, external and internal dielectrics, solvent probe radius, as well the remaining parameters for the Poisson–Boltzmann equation approximation. In sequence, APBS was used to estimate the surface electrostatic potential along the protein surface according to the linearized numerical approximation of the differential Poisson–Boltzmann equation, the original differential equation depicted in (2):

(2) $$\nabla^{2\psi }\;=-\frac{c_{0}\;\beta }{\epsilon {\;}_{sol}{}_{v\;}.^{\;}\epsilon {\;}_{sol}}\left[e^{\frac{-\beta \psi \left(x,y,z\right)}{k{\;}_{B}T}}-e^{\frac{\beta \psi \left(x,y,z\right)}{k{\;}_{B}T}}\right]$$

where *ψ* is the electric three-dimensional potential along the solute surface, $c_{0}\;$is the solvent ionic concentration (here considered at the default of 0.15 M), ε_solv_ and ε_sol_ are the respective dielectric values of the solvent (here considered at the default of 78.54) and of the solute (i.e., the protein, here considered at the default of 2), *β* is the absolute value of the charge of an electron (1.602 × 10^−19^ coulombs).

In order to be self-consistent, the atomic charges were attributed according to the same AMBER force fields previously used on the MD simulations (ff99SB for the protein and GLYCAM06 at the single calculation made for glucose) and the same protonation states were equally preserved. The remaining PBSA parameters were taken to APBS calculations by the software default.

The PBSA maps generated by the grid files were analyzed as images at the VMD software [49]. To analyze the electrostatic distribution in a comparative approach, the electrostatic maps were superposed at the respective structures using a color scale in the range of −20.00: 0.00: 20.00 for the dimensionless surface electrostatic potential *ψ* in all the systems.

### 4.6. Grid Inhomogeneous Solvation Theory Analyses

The structural water occupancy and energetics around the active site was analyzed by the grid inhomogeneous solvation theory (GIST) of the representative conformers of the minima obtained from the FEL analysis. The concept of GIST is briefly outlined in this section. For a more detailed discussion of the theoretical background, we recommend the studies described in [40,50,51]. For a better glimpse of the state of the art concerning the recent usability of this technique in different and complex biochemical systems, accurate examples can be found in [50,52,53].

Basically, GIST is a method to calculate the position-dependent free energy of the water molecules in a system on a grid-based approach. The method is made in combination with molecular dynamics simulation and takes into consideration only the phase space of the water molecules, restraining the solute to a single conformation. As this is a rough simplification of a mobile solute, multiple GIST calculations can be used to estimate the free energy of solvation to different conformers q of this same solute ∆G_solv_(**q**). The final results according to the differential probability of each conformer p(**q**) by the summation (3):

(3) $$\Delta G{\;}_{solv}\approx \Sigma \Delta G{\;}_{solv}(q)p(q)$$

The objective of the GIST calculations was to compare the differences in the energetic behavior of the water along the extension of the protein surface at the active pocket. This aimed to estimate the contribution of this same water energetic differences on the accessibility of the ligand to migrate from one subsite of the active pocket to the other as well from each subsite to the bulk or vice-versa (looking for possible paths of ligand escaping or retention based on the water exclusion principle). In this way we did not integrate the grid estimated ΔG_sol_ values, analyzing the results on a per voxel approach, i.e., we estimated the free energy of water molecules transfer from the bulk to different subsites of the studied proteins at different decoys from their respective FELs. These subsites, in turn, are computationally described as different voxels v. This per voxel ΔG_sol_ value (here called ΔG^v^_sol_), in turn, can be naturally split in two respective enthalpic and two respective entropic per voxel terms as shown in (4):

(4) $$\Delta G{\;}^{v}{\;}_{solv}=\;\Delta E{\;}^{v}{}_{sw}+\;\Delta E{\;}^{v}{\;}_{ww}-\;T\Delta S^{v}{}_{trans}-\;T\Delta S^{v}{}_{orient}$$

where the enthalpic terms Δ_sw_ and ΔE_ww_ are the respective solute–water and water–water interaction energies per voxel, as calculated by the molecular mechanics’ force field. The solvation entropy terms, in turn, encompass the respective contributions of the translational ΔS_trans_ and orientational (i.e., angular) Δ S_orient_ of the water molecules around the different voxels, defined as Shannon entropies and algorithmically calculated via the nearest neighbor estimats and orientational (i.e., angular) *Δ* S_orient_ of the water molecules around the different voxels, defined as Shannon entropies and algorithmically calculated via the nearest neighbor estimation [42,52,53,54]. All terms depicted in (4) are state functions, and therefore, need a reference state. In our case, the simulation of pure water model TIP3P was done without any solute. The reference value for the water–water interaction is, therefore, −9.533 kcal/mol, whereas the reference values for the remaining terms ΔE_SW_, ΔS_trans_, ΔS_orient_ are zero. At the specific case of the Δ E^v^_ww_, with a non-zero reference value, and considering our per voxel approach, it is also necessary to consider how much each voxel is populated on average by water molecules along the simulation frames compared to the pure water. In this way, the reference water–water interaction in each voxel E^v^_ww_(Ref) was calculated multiplying the respective numerical values of the reference energy in units of kcal/mol on a simple water–water interaction (−9.533) by the reference water density in units of water molecules/Å^3^ on a pure TIP3P simulation (0.0329) and the average per frame number density of water oxygen centers found in each voxel compared to the bulk density (g^v^(O) in units of Å^3^/0.0329 molecules), resulting in the equality 5:

(5) $$E{\;}^{v}{\;}_{ww}\left(Ref\right)\;=\;-0.3136\;g{\;}^{v}\left(O\right)\;kcal/mol$$

We have carried out the GIST analyses by the algorithm implemented at the cpptraj tool of the most recent version of the AmberTools package [36,55]. To proceed with the calculations of the respective total values of Δ E^v^_sw_, Δ E^v^_ww_, Δ S^v^_trans_, and Δ S^v^_orient_ from the density-weighted values firstly returned by the GIST tool from AMBER, as well to combine these values in order to obtain the ∆G^v^_solv_ values, we used the GISTPP tool [55]. The protocol used was that described in the same reference, as well as in the related tutorial provided by the authors’ website. The only addendum was the procedure to subtract the per voxel water–water interaction reference E^v^_ww_(Ref) estimated according to (5) in order to obtain the correctly bulk referenced Δ G^v^_sol_ values.

Looking for a higher simplification of the analysis, in all the calculations, only the protein was considered in the GIST preparative MDs and the GIST calculations. In this way, the effect of the carbohydrate ligand was considered as responsive to the water energetic effects occasioned by the protein macro-environment. Although this approach carries the error of depreciate possible modulations of the local water energetic by the ligand (overall dealing with hygroscopic ligands as carbohydrates), to introduce a rigid carbohydrate (being a naturally high flexible ligand) on the GIST designed MDs, as well on the GIST calculations themselves, could introduce more local artifacts than accuracy. Besides this, it is reasonable to suppose, overall on the semi-quantitative per site analysis here carried out, that the protein macro-environment will provide a more deterministic influence on the water energetics and that the small glucose or cellobiose molecule will respond to that. 

The reference solvent density (as mentioned above) was considered as 0.0329 water molecules/Å^3^ (adequate to the TIP3P model), and the default value of 0.5 Å was used for the grid size (volume of 0.125Å^3^ for each voxel). For all the GH1 conformers, analyses were carried out at the same box containing grid dimensions equal to 70, 90 and 80 at the respective X Y Z axes. Analogously, the GIST analyses were carried out at the same 90, 90, 90 grid box for all the GH3 conformers. In both cases, the box was placed in such a way to encompass the catalytic residues, the +1 and -1 sites, as well the +2 one in the case of the GH1 enzyme, beyond all the extension of the exit channel and loops around. 

Finally, for the analysis of the significantly favorable or unfavorable (i.e., constrained) hydration regions, only the sites containing water molecules considerably retained (respective g^v^(O) values ≥ 10 units of the bulk density) and with ∆G^v^_solv_ values respectively ≤ −3 kcal^.^mol^−1^ or ≥ 3 kcal^.^mol^−1^ were considered.

## 5. Conclusions 

We used molecular dynamics simulations to understand the behavior of glucose and cellobiose in a GH1 (HiBG) and a GH3 (AaBG) β-glucosidase. We hypothesize that the explanation for glucose tolerance is related to a sophisticated mechanism of ligand release by the substrate channel, herein called “slingshot mechanism”, that affects glucose more than cellobiose in GH1 enzymes. The proposed mechanism is in agreement with previous studies [3,12,13,25], confirming that the shape of the substrate channel of glucose-tolerant enzymes, the pharmacophoric properties of amino acids, the affinity of glucose by allosteric sites, and the protein motion act together to allow the glucose tolerance. It suggests that ligands in glucose-tolerant β-glucosidases are guided to a hydrophobic bottleneck region in the entrance of the substrate channel and released by interactions with a hydrogen bond acceptor amino acid residue present in this region (D238 in HiBG, Videos S1 and S2). Furthermore, the dynamics of favorable and unfavorable waters, the electrostatic distribution, as well as the product/substrate fitting relationship contribute to explain the glucose tolerance of GH1 β-glucosidases and the non-tolerance of the GH3s. The results presented in this paper open new pathways for elucidating the mechanisms of tolerance and inhibition by the product. Beyond that, they give new insights for the rational design of new enzymes resistant to inhibition and, consequently, the viability of the large-scale production of biofuels.

## Supplementary Materials

The following are available online at https://www.mdpi.com/1420-3049/24/18/3215/s1. Supplementary PDF file (Figures S1–S13, Tables S1–S6). Video S1: MD of the glucose exit in a glucose-tolerant GH1 β-glucosidase (also available at <https://youtu.be/fzWynXUbdcI>). Video S2: Interactions among glucose, D228, K257, and N312 (also available at <https://youtu.be/7VzR0CVag6I>).
