# Supplementary material for: Molecular Dynamics Gives New Insights into the Glucose Tolerance and Inhibition Mechanisms on β-Glucosidases
Source: Molecules. 2019 Sep 4;24(18):3215. doi: 10.3390/molecules24183215 (PMC6766793; doi:10.3390/molecules24183215)
Supplement: Supplementary file 1 [file molecules-24-03215-s001.pdf]

Supplementary Material for "Molecular dynamics gives new insights into the glucose tolerance and inhibition mechanisms on β-glucosidases"

Leon Sulfierry Corrêa Costa [1,†], Diego César Batista Mariano [2,†], Rafael Eduardo Oliveira Rocha [2,3], Johannes Kraml [4], Carlos Henrique da Silveira [5], Klaus Roman Liedl [4], Raquel Cardoso de Melo-Minardi [2] and Leonardo Henrique Franca de Lima [1,*]

[1] Laboratory of Molecular and Bioinformatics Modeling, Department of Exact and Biological Sciences (DECEB), Universidade Federal de São João Del-Rei, Campus Sete Lagoas, 35701-970 Sete Lagoas, Brazil
[2] Laboratory of Bioinformatics and Systems (LBS), Department of Computer Science, Universidade Federal de Minas Gerais, 31270-901 Belo Horizonte, Brazil
[3] Laboratory of Molecular Modeling and Drug Design, Department of Biochemistry and Immunology, Universidade Federal de Minas Gerais, 31270-901 Belo Horizonte, Brazil
[4] Institute of General, Inorganic and Theoretical Chemistry (IGITC), Center for Molecular Biosciences Innsbruck (CMBI), Leopold-Franzens-Universität-Innsbruck, Innrain 82, 6020 Innsbruck, Austria
[5] Institute of Technological Sciences, Universidade Federal de Itajubá, Campus Itabira, 35903-087, Itabira, Brazil
* Correspondence: leofrancalima@ufsj.edu.br; Tel.: +55-31-3775-5500
† These authors contributed equally to this work

Fig. S1. The two starting poses for glucose along the GH1 simulations and comparison with different crystallographic structures. (A) Comparison of the crystallographic pose of glucose at the PDB:4MDP (HiBG) in green (between subsites +1/+2) and the poses usually found at other GH1s (at the -1 subsite). In blue at PDB:4PTX, in yellow at PDB:2JIE, in wheat at PDB:2E40, in magenta at PDB:3WH6 and in pale green at PDB:2O9T. The orange spheres depict the hydrophobic side chains packed against glucose in PDB:4MDP. The red sticks depict the two catalytic acids in subsite -1; (B) Comparison of the PDB:4MDP pose (green) with the PDB:4PTX one (cyan), depicting the usual high electron density (considering $\sigma = 1.5$) around glucose at the -1 subsite. At this position is the starting pose of the simulation. (C) Sparse electron density around glucose at the +1/+2 subsites in PDB:4MDP ($\sigma = 1.5$), and higher one around glycerol (yellow) found at the -1 subsite at the same structure. (D) Structural superposition of the glycerol found in PDB:4MDP (yellow) and the glucose in PDB:4PTX (cyan), both at the same -1 subsite.

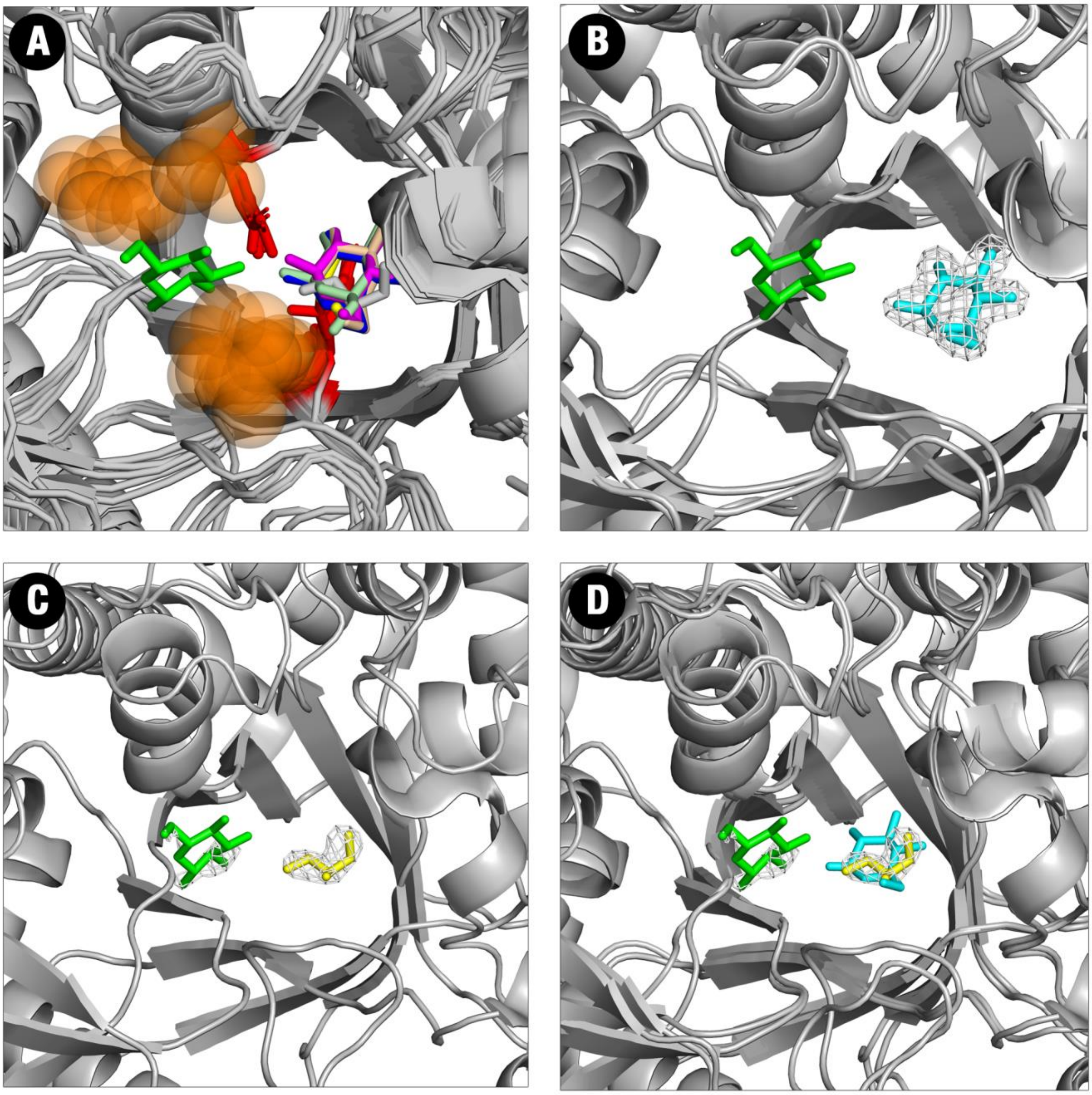

Fig. S2. Structural alignment of representative poses of HiBG in complex with glucose (GH1-Glucose). The superposed images represent the different FEL regions in Figure 2B and illustrate the different poses in Figure 3. The structure is shown in gray cartons; ligand in green sticks; subsite -1 in red sticks; subsite +1/+2 in yellow sticks; and residues W169 (orange sticks), D238 and W350 (blue sticks).

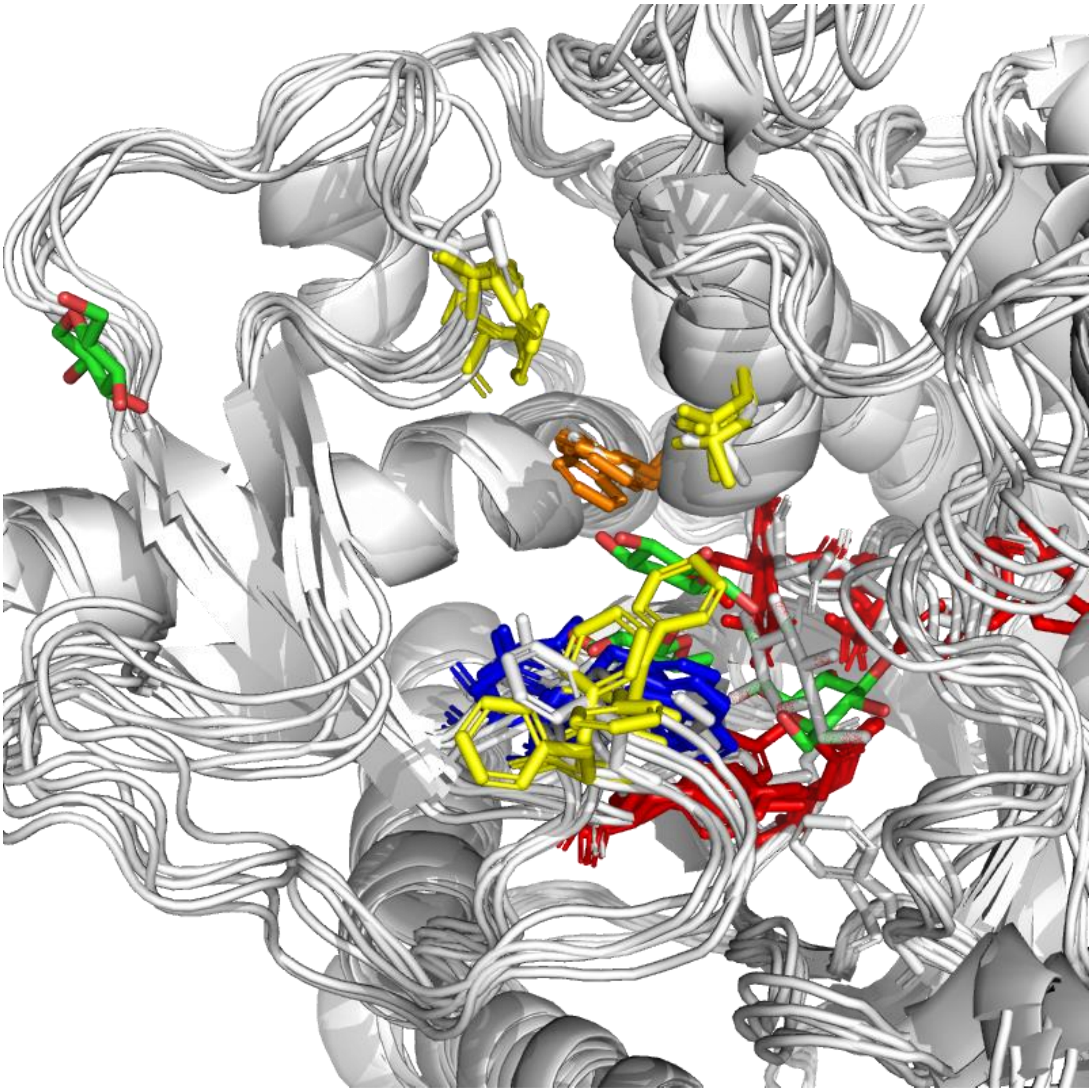

Fig. S3. Structural alignment of representative conformations of HiBG in complex with cellobiose (GH1-Cellobiose). The superposed images represent the different FEL regions in Figure 2A. The structure is shown in gray cartons; ligand in green sticks; subsite -1 in red sticks; subsite +1/+2 in yellow sticks; and residues W169 (orange sticks), D238 and W350 (blue sticks).

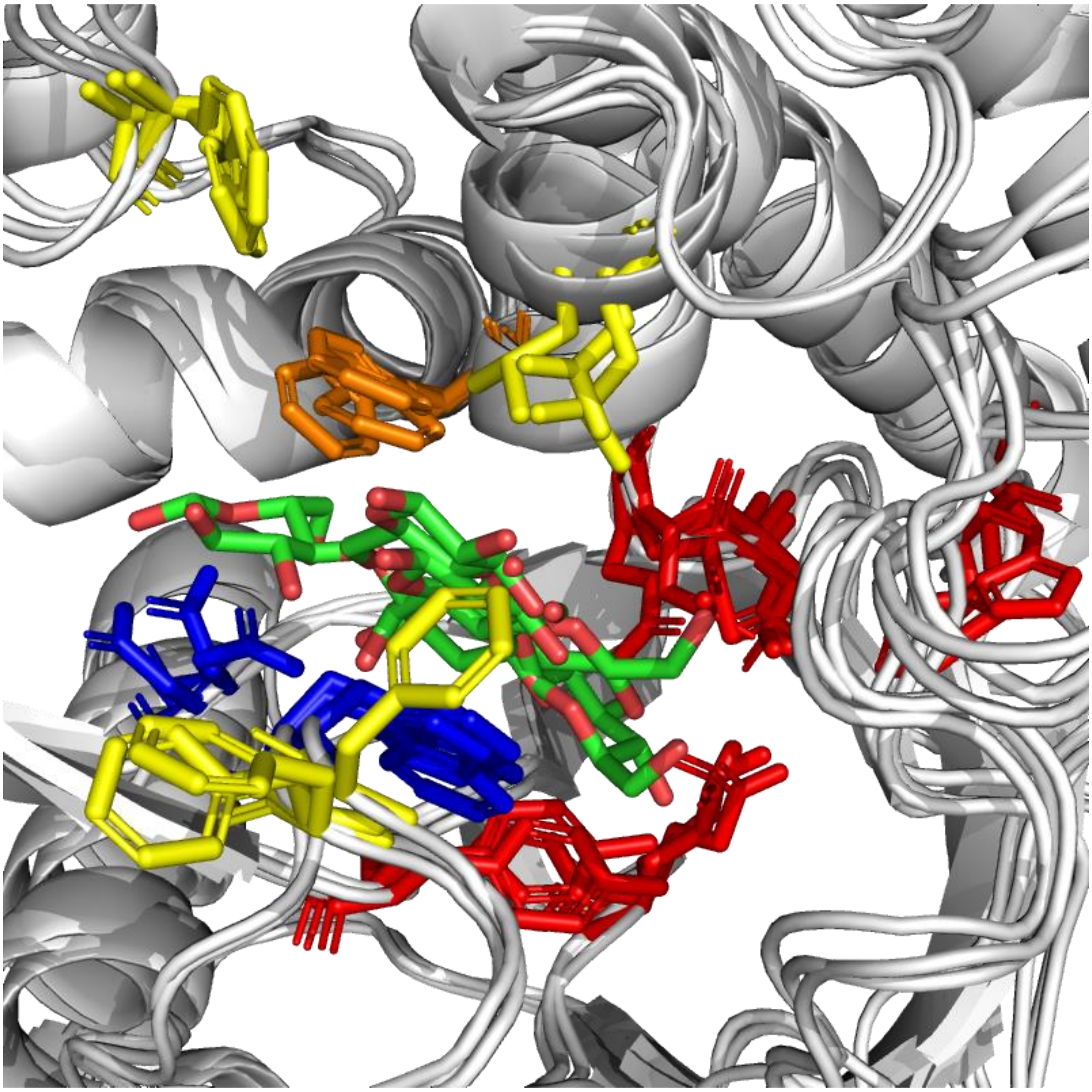

Fig. S4. Structural alignment of representative conformations of AaBG in complex with glucose (GH3-Glucose). The superposed images represent the different FEL regions in Figure 2D. The structure is shown in gray cartons; ligand in green sticks; subsite -1 in red sticks; and subsite +1/+2 in yellow sticks.

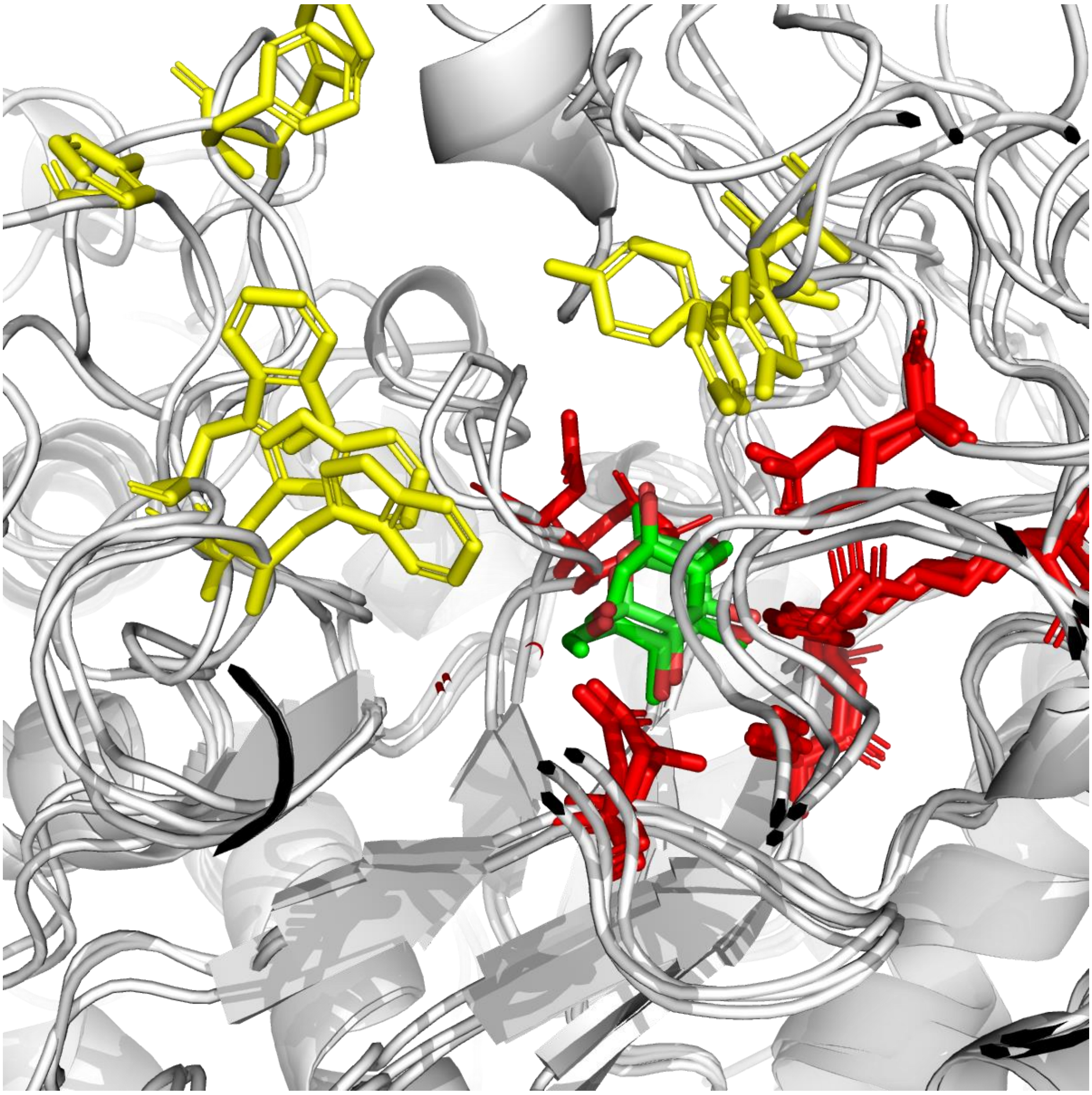

Fig. S5. Structural alignment of representative conformations of AaBG in complex with cellobiose (GH3-Cellobiose). The structure is shown in gray cartons; ligand in green sticks; -1 subsite in red sticks; and +1/+2 subsite in yellow sticks.

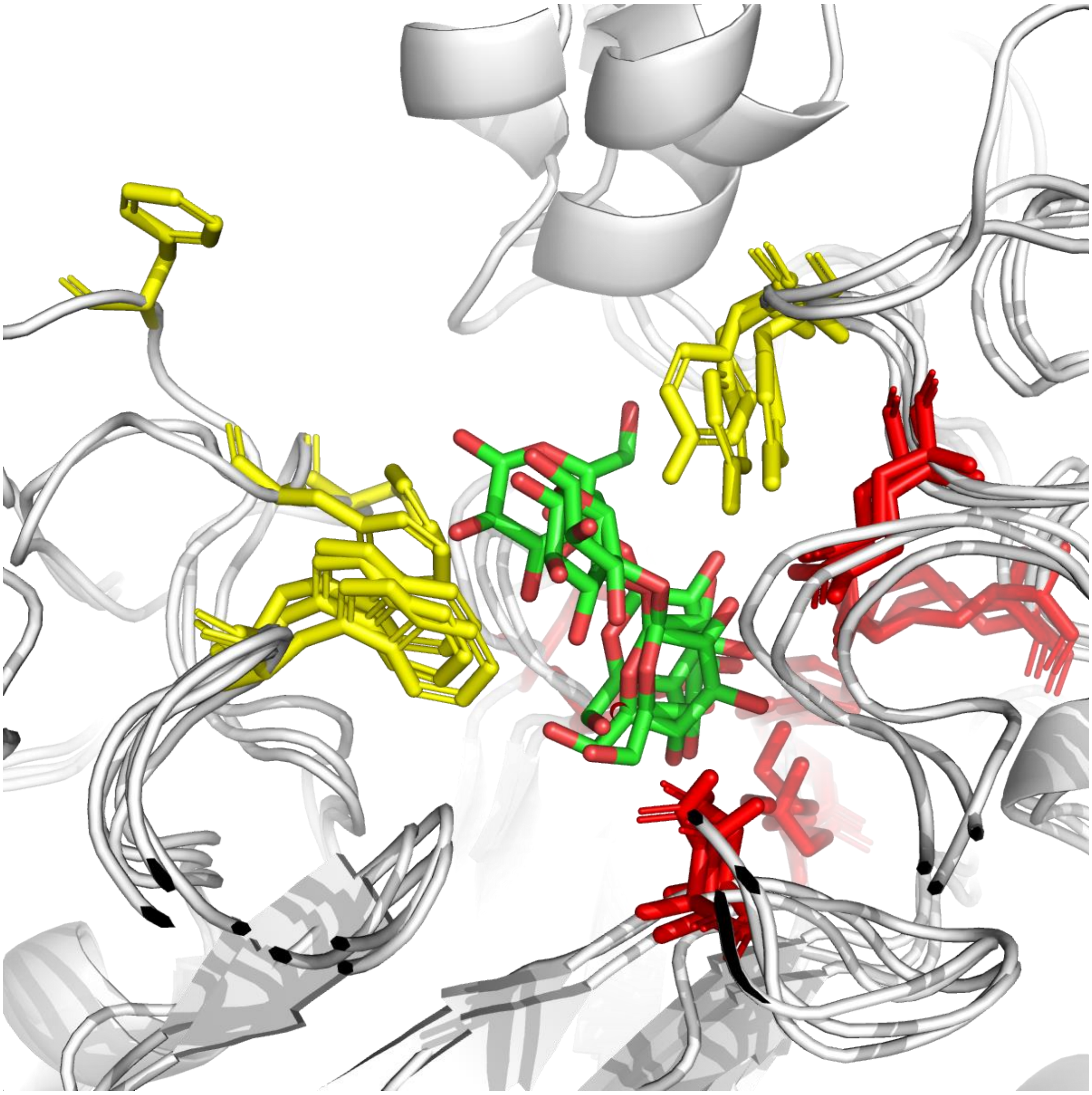

Fig. S6. Statistics for the ligand-protein contacts and structure of the substrate channel in GH1-cellobiose complex. The FEL depicted in Figure 2A was spliced in two representative blocks: B1, corresponding to the region 1 (more internalized cellobiose, accounting for 49 % of the data) and B2, corresponding to the sum of regions 2 and 3 (cellobiose interacting with the hydrophobic bottleneck and with AS, accounting for 51 % of the data). Contacts estimated by the LIGPLOT software (https://www.ebi.ac.uk/thornton-srv/software/LIGPLOT). Just the direct hydrogen bonds and the hydrophobic interactions are depicted. Interactions involving reducing (R) and non-reducing (nR) glucose extremities are treated separately. Bottom: A draft of the different sub-regions of the active pocket is depicted. The smaller figure illustrates this same draft superposed to the protein structure in the cartoon. Section 1 contains the residues that the ligand has interacted on our MD sets along its exit path. Cyan apolar residues, pale green negative residues, magenta positive residues, black polar neutral residues.

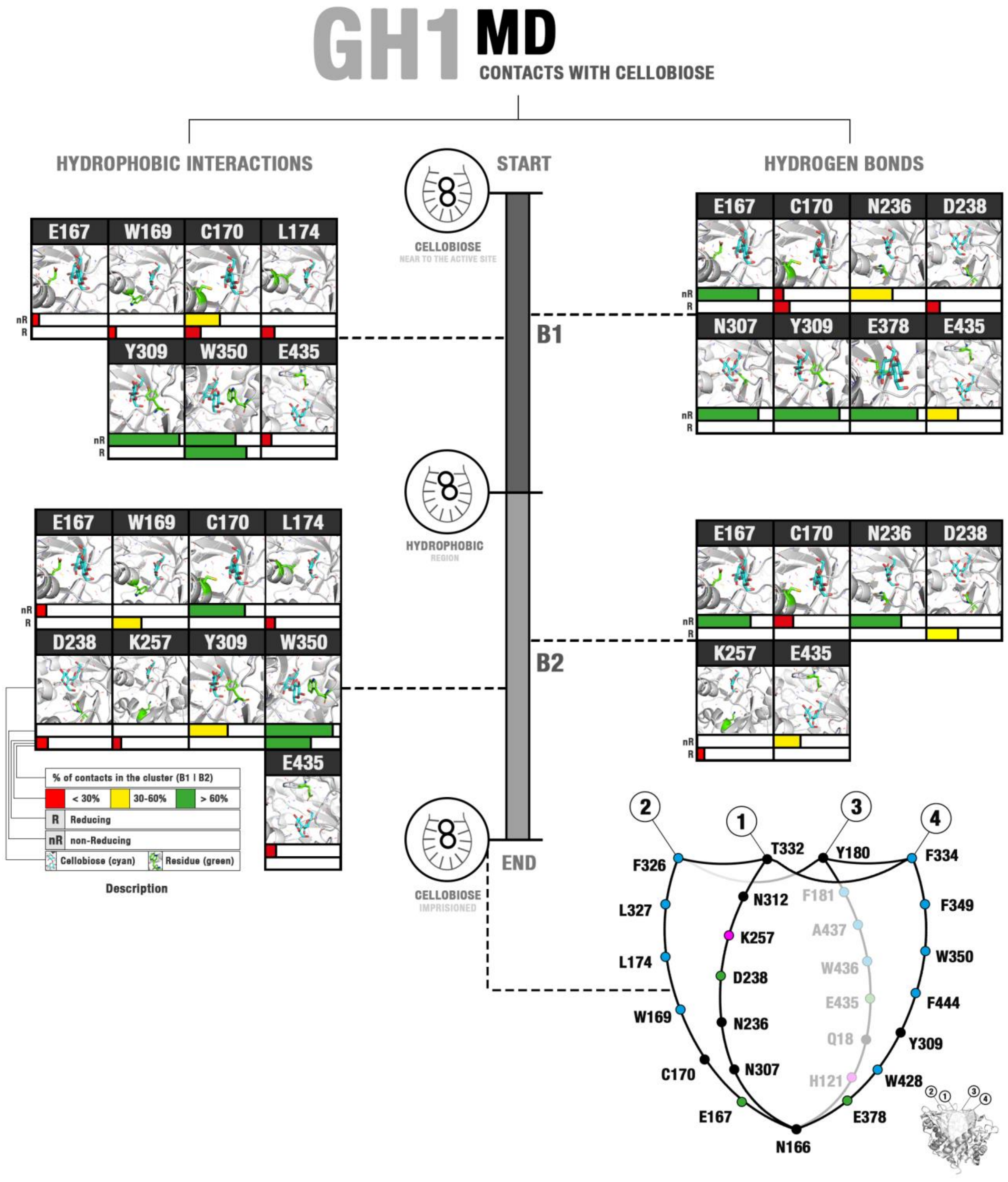

Fig. S7. Statistics for the ligand-protein contacts and structure for the substrate channel in GH1-Glucose complex. The FEL depicted in Figure 2B was spliced in three representative blocks: B1, corresponding to the region 1 (catalytic cleft, accounting for 18 % of the data at the 1-5 FEL region), B2, corresponding to the region 2 (middle of the substrate channel accounting for 58 % of the same data) and B3 corresponding to the regions 3, 4 and 5 (hydrophobic bottleneck, allosteric site and exit, accounting for 24 %). Region 6, in that glucose is outside the protein, was not considered. Contacts were estimated by the LIGPLOT software (https://www.ebi.ac.uk/thornton-srv/software/LIGPLOT). Only the direct hydrogen bonds and the hydrophobic interactions are depicted. Interactions involving reducing (R) and non-reducing (nR) glucose extremities are treated separately. At the bottom and the right, a draft of the different sub-regions of the active pocket is depicted. The smaller figure illustrates this draft superposed to the protein structure in the cartoon. Section 1 contains the residues that the ligand has interacted on our MD sets along its exit path. Cyan apolar residues, pale green negative residues, magenta positive residues, black polar neutral residues.

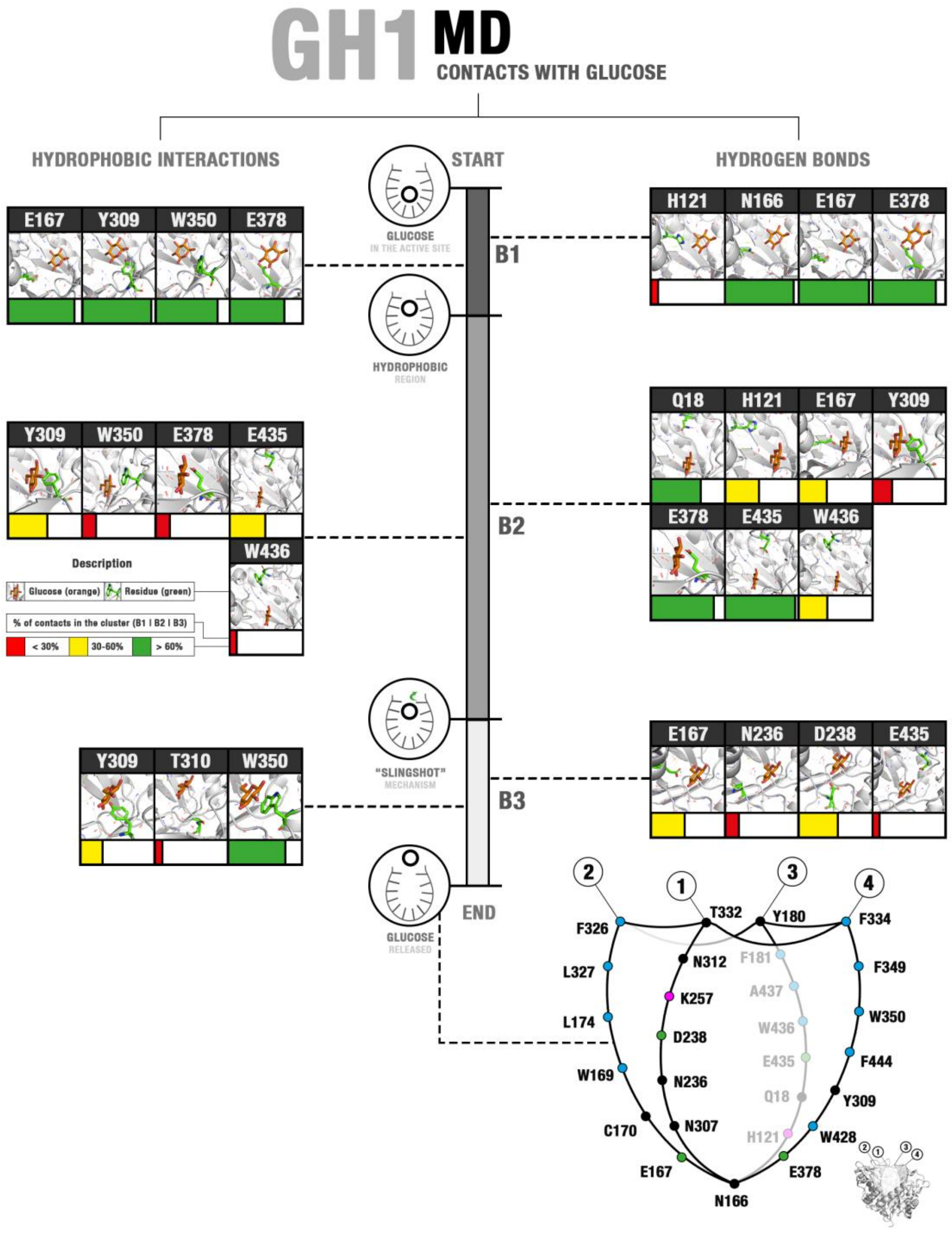

Fig. S8. Statistics for the ligand-protein contacts estimated along the MD sets for the GH3-Cellobiose complex. The FEL depicted in Figure 2C was considered integrally due to the significant lower ligand mobility at this system. Contacts estimated by the LIGPLOT software (https://www.ebi.ac.uk/thornton-srv/software/LIGPLOT). Only the direct hydrogen bonds and the hydrophobic interactions are depicted. Interactions involving reducing (R) and non-reducing (nR) glucose extremities are treated separately.

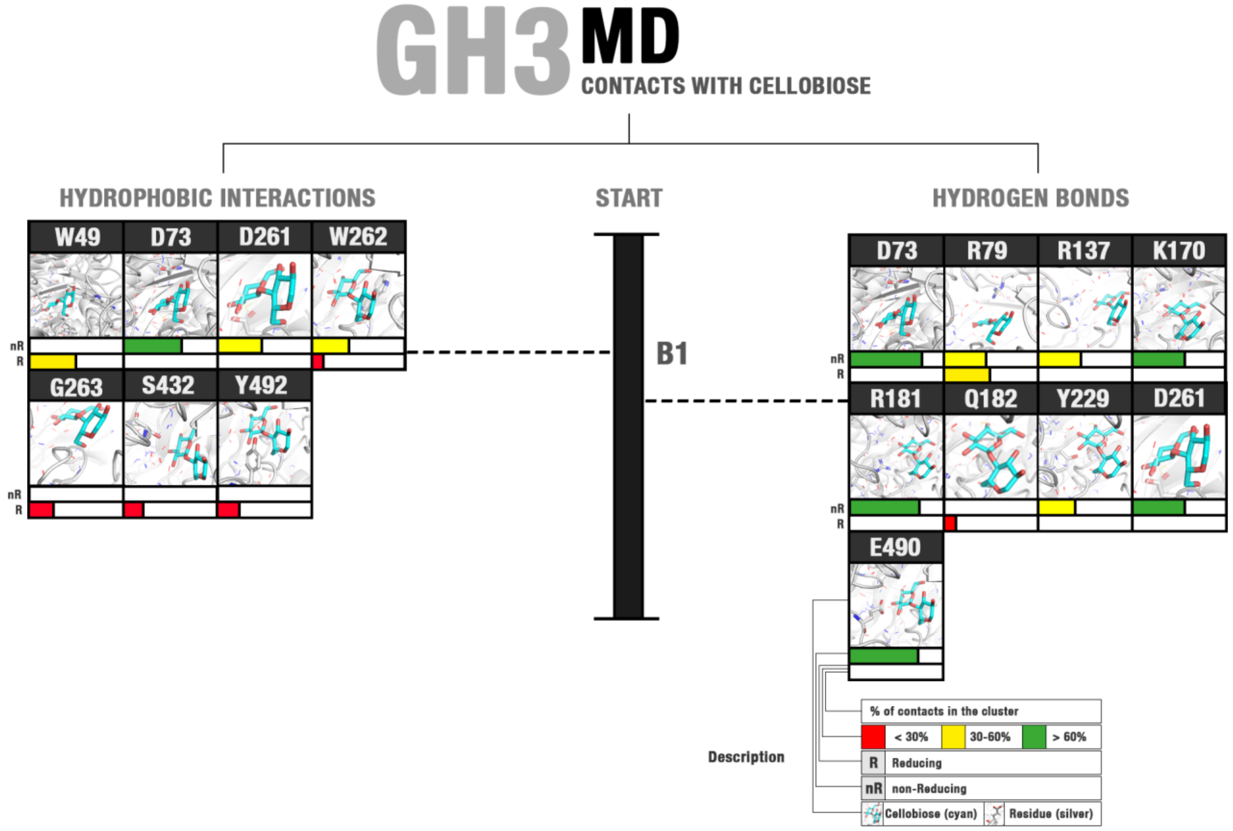

Fig. S9Statistics for the ligand-protein contacts estimated along the MD sets for the GH3-Glucose complex. The FEL depicted in Figure 2D was considered integrally due to the significant lower ligand mobility at this system. Contacts estimated by the LIGPLOT software (https://www.ebi.ac.uk/thornton-srv/software/LIGPLOT). Only the direct hydrogen bonds and the hydrophobic interactions are depicted. Interactions involving reducing (R) and non-reducing (nR) glucose extremities are treated separately.

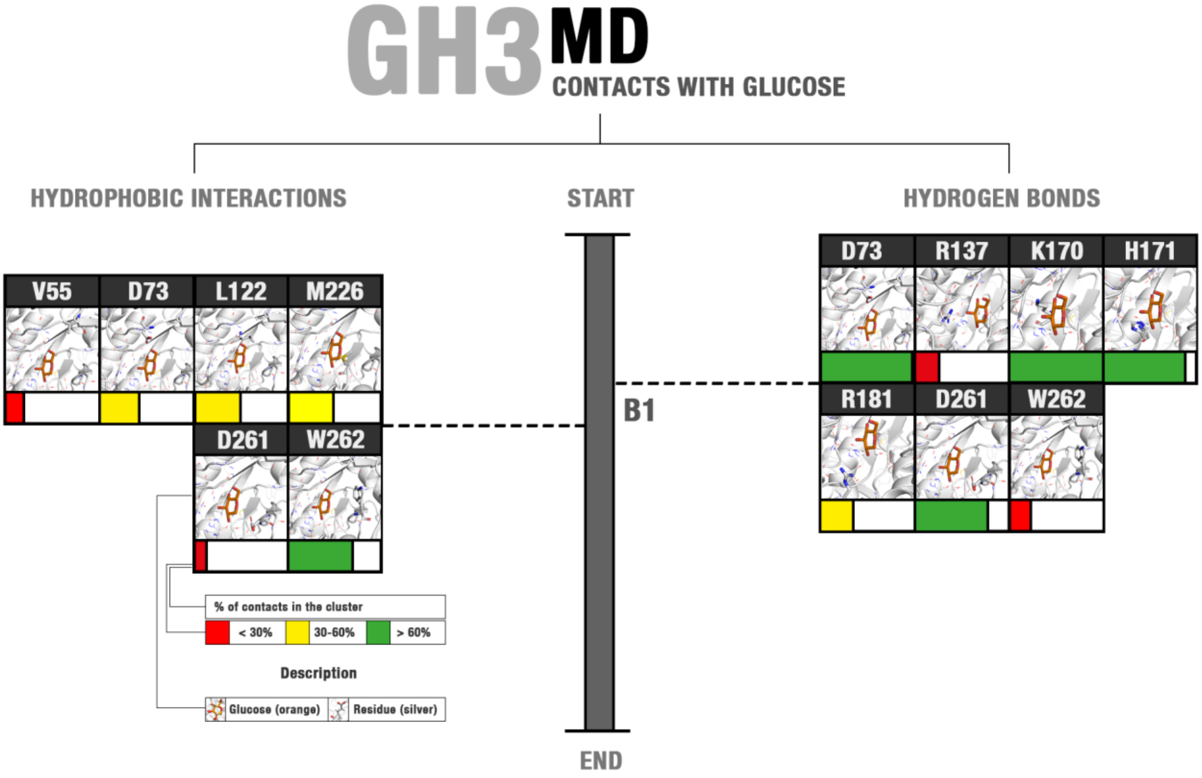

Fig. S10. Comparison of the principal components for the protein and ligand in the different systems. In all analysis, the entire set of frames were aligned to the average structure considering the protein backbone atoms (Cα, C, O, N) along all the set of simulations. In *blue* and *red* are depicted the respective first and the second principal component of the protein movements (described mainly by the movements of the loops around the active site). In black it is shown the first principal component of the ligand (considering the translational, rotational and vibrational movements) related to the protein. For the GH1-glucose MD sets, there were not considered the frames at the region 6 of the FEL depicted in Figure 2B (with the ligand already outside the protein). It can be noted the higher ligand motion in GH1, despite the higher amplitude of the loop movements in GH3. It can also be noted the significantly higher response of the ligand movements to that from the protein in GH1 than in GH3.

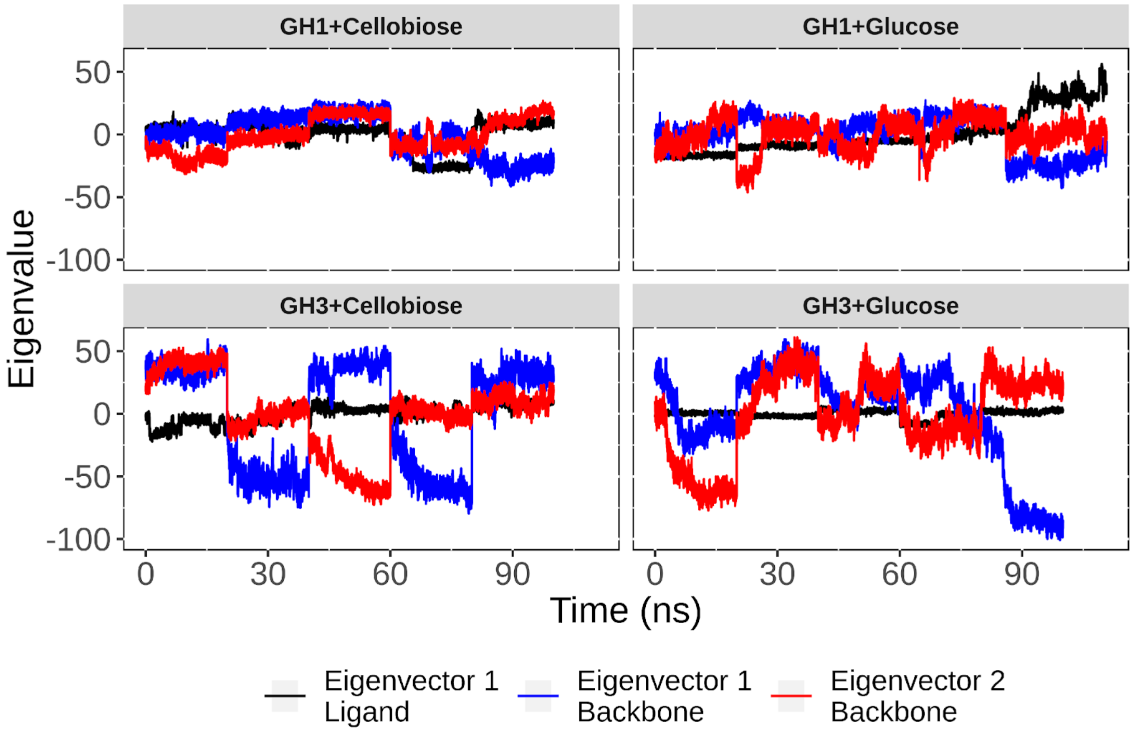

Fig. S11. Results of APBS and GIST for HiBG and AaBG.

(A-C) GH1; and (D-F) GH3.

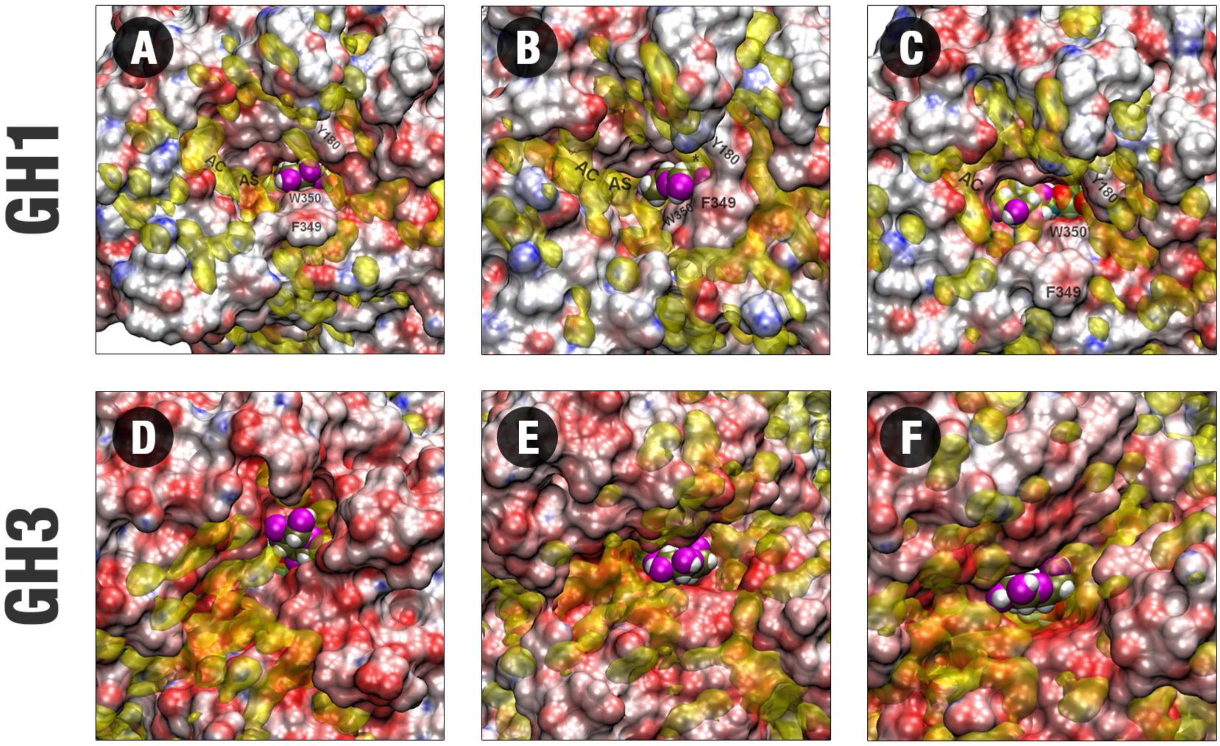

Fig. S12. RMSD against the first frame of each trajectory shows convergence for all systems.

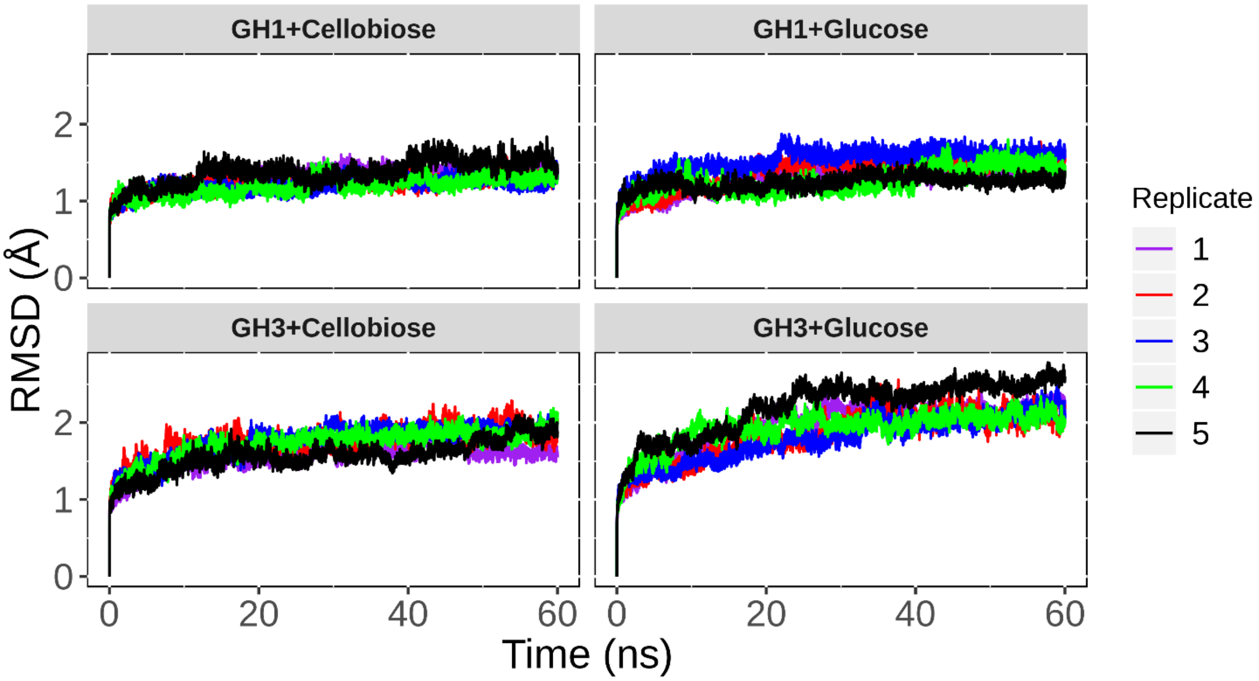

Fig. S13. Conformations recovered by the FEL profiles of the glucose positioning in GH1 and GH3, colored by the APBS and GIST data. (A-F) APBS and GIST results and glucose positions occupancy at the respective FEL regions 1-4 and two different samples of region 5 in GH1 at Figure 2B. (G-I) The analog profiles for the FEL respective regions 1-3 in GH3 at Figure 2C. APBS colors are used just for the protein potential in A, C-I and both for the protein as the glucose potential in B. APBS scale in red-white-blue corresponding to ψ values of -20.00:0.00:+20.00, respectively. GIST positions are shown in yellow transparent surfaces. Glucose is shown in spheres in all the figures and, not accounting in B (as previously mentioned), is colored with the carbons in cyan, the oxygens in red and the hydrogens in white. Residues involved in the establishment of the hydrophobic cage and regions important for the glucose escaping in GH1 are highlighted. AS - Allosteric site. AC - Allosteric channel. A white asterisk (*) is used to point the site for water exclusion confined by the hydrophobic cage between Y180, F349, and W350.

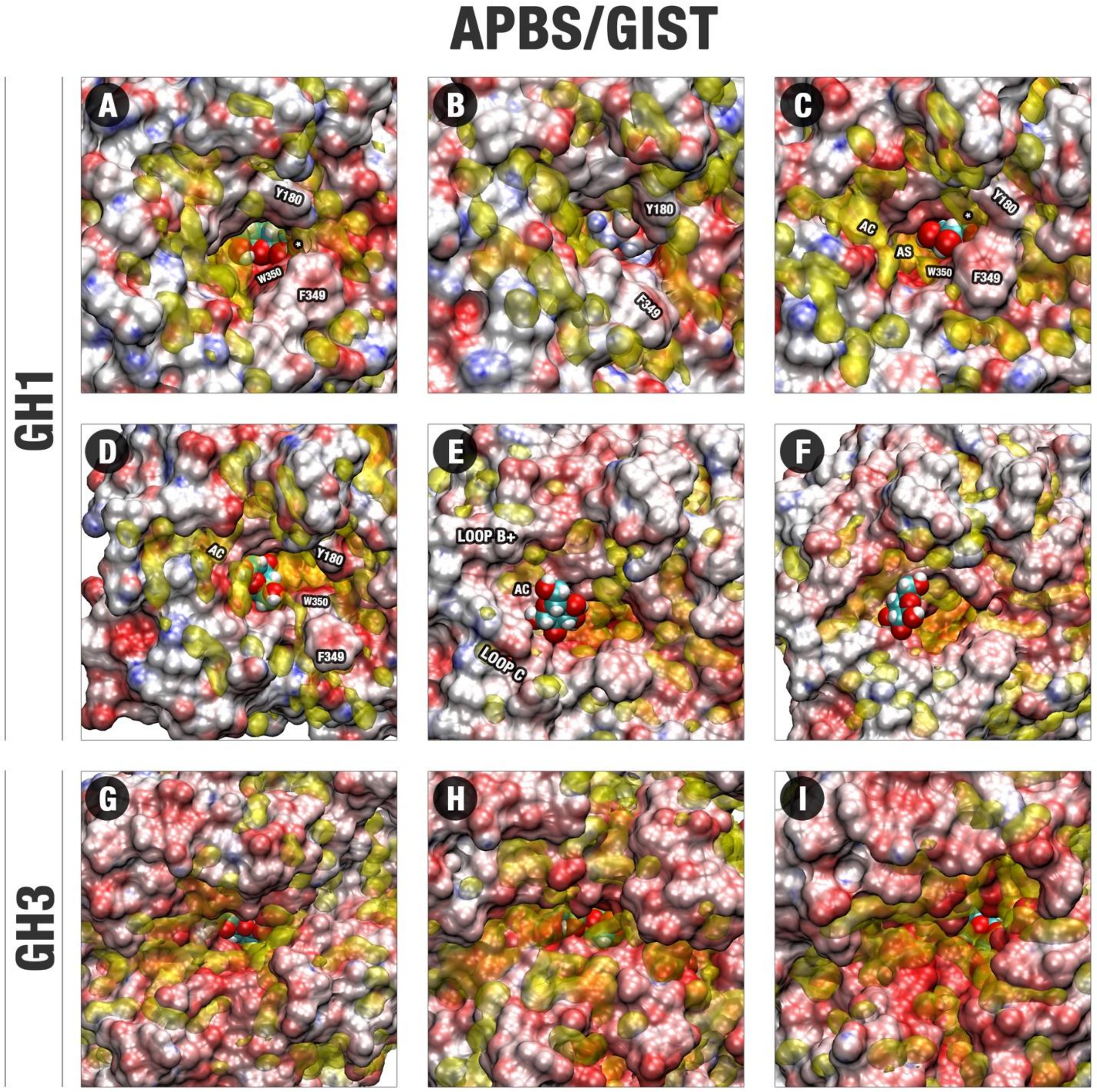

Table S1. Percentage of hydrogen bonds between residues of HiBG (GH1) with glucose (G) and cellobiose (C).

The frames were divided into three blocks for the glucose MD sets (G-B1, G-B2, and G-B3) and two blocks for cellobiose (C-B1 and C-B2), according to previously described in Fig. S6-7. Representativeness (considering all replicates): G-B1 (18%), G-B2 (58%), G-B3 (24%), C-B1 (46%), and C-B2(48%). Blocks with contacts lower than 10% were not considered. Polar contacts involving the reducing -OH were considered separately. For cellobiose, the contacts involving the respective reducing (R) and non-reducing (nR) glucose rings are also considered separately. Contact analysis was calculated using LIGPLOT (https://www.ebi.ac.uk/thornton-srv/software/LIGPLOT).

	**G-B1**	**G-OH-B1**	**G-B2**	**G-OH-B2**	**G-B3**	**G-OH-B3**	**C-NR-B1**	**C-R-B1**	**C-OH-B1**	**C-NR-B2**	**C-R-B2**	**C-OH-B2**
**Q18**			68.74									
**H121**	12.12		47.64									
**W122**												
**N166**	99.8											
**E167**		97.38	32.93	39.23	36.15	12.15	81.26			70.95		
**W169**												
**C170**							15.22	23.01		27.92		
**L174**												
**T178**												
**Y180**												
**N236**					21.05		54.77			68.44		
**G237**												
**D238**					54.1			19.55			34.23	27.23
**K257**											15.53	
**N307**		98.2					81.66					
**Y309**				29.37			87.11					
**T310**												
**N312**												
**T332**												
**S348**												
**W350**												
**E378**	87.74		73.94	31.23			88.64					
**N433**												
**E435**			94.21		12.96		43.74			37.14		
**W436**			42.39									
**A437**												
**E438**												

Table S2. Percentage of water-mediated hydrogen bonds between residues of HiBG (GH1) with glucose (G) and cellobiose (C).

The frames were divided into three blocks for the glucose MD sets (G-B1, G-B2, and G-B3) and two blocks for cellobiose (C-B1 and C-B2), according to previously described in Fig. S6-7. Representativeness (considering all replicates): G-B1 (18%), G-B2 (58%), G-B3 (24%), C-B1 (46%), and C-B2(48%). Blocks with contacts lower than 10% were not considered. Polar contacts involving the reducing -OH were considered separately. For cellobiose, the contacts involving the respective reducing (R) and non-reducing (nR) glucose rings are also considered separately. Contact analysis was calculated using LIGPLOT (https://www.ebi.ac.uk/thornton-srv/software/LIGPLOT).

	**G-B1**	**G-OH-B1**	**G-B2**	**G-OH-B2**	**G-B3**	**G-OH-B3**	**C-NR-B1**	**C-R-B1**	**C-OH-B1**	**C-NR-B2**	**C-R-B2**	**C-OH-B2**
**Q18**	90.18		29.87		14.84		45.01			10.74		
**H121**	26.77											
**W122**	79.79											
**N166**												
**E167**	99.68	41.88	60.61	30.34	52.89	11.86	34.24	14.08		34.99		
**W169**												
**C170**	40.72		12.56	15.61	20.86					11.32		
**L174**												
**T178**											19.14	
**Y180**								39.63			44.02	
**N236**	37.13	26.17		16.56	32.45			17.37		29.26	12.37	
**G237**					26.26					32.32		
**D238**					52.02			39.28			42.8	
**K257**					13.82						14.6	
**N307**			28.93				39.21			20.03		
**Y309**	22.53				36.68					39.82		
**T310**					31.51					27.25		
**N312**											39.07	
**T332**											23.5	
**S348**			10.79									
**W350**								12.47		12.56	37.73	
**E378**	76.35		37.33	12.49	14.77		33.53			42.78		
**N433**					10.25							
**E435**	28.93		45.76				76.34	62.15		74.87		
**W436**	30.35									12.02		
**A437**			10.08									
**E438**			15.29					41.2		20.23	46.08	

Table S3. Percentage of hydrophobic contacts between residues of HiBG (GH1) with glucose (G) and cellobiose (C).

The frames were divided into three blocks for glucose (G-B1, G-B2, and G-B3) and two blocks for cellobiose (C-B1 and C-B2). Representativeness (considering all replicates): G-B1 (18%), G-B2 (58%), G-B3 (24%), C-B1 (46%), and C-B2(48%). Blocks with contacts lower than 10% were not considered. For cellobiose, the contacts involving the respective reducing (R) and non-reducing (nR) glucose rings were considered separately. Contact analysis was calculated using LIGPLOT (https://www.ebi.ac.uk/thornton-srv/software/LIGPLOT).

	G-B1	G-B2	G-B3	C-nR-B1	C-R-B1	C-nR-B2	C-R-B2
**Q18**							
**H121**							
**W122**							
**N166**							
**E167**	90			12.45		16.42	
**W169**					14.46		38.92
**C170**				43.88	20.67	73.99	
**L174**					22.28		15.47
**T178**							
**Y180**							
**N236**							
**G237**							
**D238**							18.4
**K257**							14.42
**N307**							
**Y309**	95.98	54.3	32.23	94.99		49.14	
**T310**			13.48				
**N312**							
**T332**							
**S348**							
**W350**	84.27	22.26	78.35	67.41	82.21	89.33	63.27
**E378**	75.61	25.55					
**N433**							
**E435**		49.02		12.81		17.91	
**W436**		12.11					
**A437**							
**E438**							

Table S4. Percentage of hydrogen bonds between residues of AaBG (GH3) with glucose (G) and cellobiose (C). All frames were grouped into a single block for glucose (G-B0) and another one for cellobiose (C-B1). Contacts with representativity lower than 10% were not considered. Polar contacts involving the reducing -OH were considered separately (for cellobiose, direct hydrogen bonds involving this group were not verified with statistic equal or higher 10 %). For cellobiose, the contacts involving the respective reducing (R) and non-reducing (nR) glucose rings are also considered separately. Contact analysis was calculated using LIGPLOT (https://www.ebi.ac.uk/thornton-srv/software/LIGPLOT).

	G-B0	G-OH-B0	C-nR-B1	C-R-B1
**W49**				
**V55**				
**D73**	99.96		82	
**R79**			41.56	52.06
**L122**				
**R137**	25.18		40.15	
**E140**				
**K170**	98.78		62.44	
**H171**	89.64			
**E178**				
**R181**	36		71.38	
**Q182**				13.64
**E185**				
**Y189**				
**M226**				
**Y229**			32.66	
**D261**	77.89	34.21	60.32	
**W262**		19.75		
**G263**				
**H265**				
**T285**				
**F286**				
**D418**				
**S432**	10.72			
**G433**				
**E490**			77.13	
**Y492**				

Table S5. Percentage of water-mediated hydrogen bonds between residues of AaBG (GH3) with glucose (G) and cellobiose (C). All frames were grouped into a single block for glucose (G-B0) and another one for cellobiose (C-B1). Contacts with representativity lower than 10% were not considered. Polar contacts involving the reducing -OH were considered separately. For cellobiose, the contacts involving the respective reducing (R) and non-reducing (nR) glucose rings are also considered separately. Contact analysis was calculated using LIGPLOT (https://www.ebi.ac.uk/thornton-srv/software/LIGPLOT).

	G-B0	G-OH-B0	C-nR-B1	C-R-B1	c-OH
**W49**					
**V55**					
**D73**	36.35		18.53		
**R79**				37.25	
**L122**					
**R137**	13.7				
**E140**			10.89		
**K170**	18.4		32.9		
**H171**	12.66		23.06		
**E178**					
**R181**	18.34	18.25			
**Q182**		24.7		12.68	
**E185**			42.23	20.96	
**Y189**					
**M226**					
**Y229**					
**D261**	24.22	64.51	26.74	12.08	
**W262**		18.69	25.88		23.11
**G263**					
**H265**					
**T285**					
**F286**					
**D418**					
**S432**	21.32			14.19	
**G433**					
**E490**	23.52	35.25	21.82		
**Y492**					

Table S6. Percentage of hydrophobic contacts between residues of AaBG (GH3) with glucose (G) and cellobiose (C). All frames were grouped into a single block for glucose (G-B0) and another one for cellobiose (C-B1). Contacts with representativity lower than 10% were not considered. For cellobiose, the contacts involving the respective reducing (R) and non-reducing (nR) glucose rings were considered separately. Contact analysis was calculated using LIGPLOT (https://www.ebi.ac.uk/thornton-srv/software/LIGPLOT).

	G-B0	C-nR-B1	C-R-B1
**W49**			47.18
**V55**	20.1		
**D73**	42.27	66.45	
**R79**			
**L122**	45.39		
**R137**			
**E140**			
**K170**			
**H171**			
**E178**			
**R181**			
**Q182**			
**E185**			
**Y189**			
**M226**	47.73		
**Y229**			
**D261**	21.15	46.9	
**W262**	74.96	39.11	12.43
**G263**			26.67
**H265**			
**T285**			
**F286**			
**D418**			
**S432**			20.05
**G433**			
**E490**			
**Y492**			23.74
